# Activity-Induced Cortical Glutamatergic Neuron Nascent Proteins

**DOI:** 10.1523/JNEUROSCI.0707-22.2022

**Published:** 2022-10-19

**Authors:** Lucio M. Schiapparelli, Yi Xie, Pranav Sharma, Daniel B. McClatchy, Yuanhui Ma, John R. Yates, Anton Maximov, Hollis T. Cline

**Affiliations:** ^1^Neuroscience Department and Dorris Neuroscience Center, Scripps Research Institute, La Jolla, California 92037; ^2^Department of Molecular Medicine, Scripps Research Institute, La Jolla, California 92037; ^3^Skaggs Graduate School, Scripps Research Institute, La Jolla, California 92037; ^4^Xosomix, San Diego, California 92121

**Keywords:** activity dependent, BONCAT, cortex, nascent protein, neuroproteomics, seizure

## Abstract

Neuronal activity initiates signaling cascades that culminate in diverse outcomes including structural and functional neuronal plasticity, and metabolic changes. While studies have revealed activity-dependent neuronal cell type-specific transcriptional changes, unbiased quantitative analysis of cell-specific activity-induced dynamics in newly synthesized proteins (NSPs) synthesis *in vivo* has been complicated by cellular heterogeneity and a relatively low abundance of NSPs within the proteome in the brain. Here we combined targeted expression of mutant MetRS (methionine tRNA synthetase) in genetically defined cortical glutamatergic neurons with tight temporal control of treatment with the noncanonical amino acid, azidonorleucine, to biotinylate NSPs within a short period after pharmacologically induced seizure in male and female mice. By purifying peptides tagged with heavy or light biotin-alkynes and using direct tandem mass spectrometry detection of biotinylated peptides, we quantified activity-induced changes in cortical glutamatergic neuron NSPs. Seizure triggered significant changes in ∼300 NSPs, 33% of which were decreased by seizure. Proteins mediating excitatory and inhibitory synaptic plasticity, including SynGAP1, Pak3, GEPH1, Copine-6, and collybistin, and DNA and chromatin remodeling proteins, including Rad21, Smarca2, and Ddb1, are differentially synthesized in response to activity. Proteins likely to play homeostatic roles in response to activity, such as regulators of proteastasis, intracellular ion control, and cytoskeleton remodeling proteins, are activity induced. Conversely, seizure decreased newly synthetized NCAM, among others, suggesting that seizure induced degradation. Overall, we identified quantitative changes in the activity-induced nascent proteome from genetically defined cortical glutamatergic neurons as a strategy to discover downstream mediators of neuronal plasticity and generate hypotheses regarding their function.

**SIGNIFICANCE STATEMENT** Activity-induced neuronal and synaptic plasticity are mediated by changes in the protein landscape, including changes in the activity-induced newly synthesized proteins; however, identifying neuronal cell type-specific nascent proteome dynamics in the intact brain has been technically challenging. We conducted an unbiased proteomic screen from which we identified significant activity-induced changes in ∼300 newly synthesized proteins in genetically defined cortical glutamatergic neurons within 20 h after pharmacologically induced seizure. Bioinformatic analysis of the dynamic nascent proteome indicates that the newly synthesized proteins play diverse roles in excitatory and inhibitory synaptic plasticity, chromatin remodeling, homeostatic mechanisms, and proteasomal and metabolic functions, extending our understanding of the diversity of plasticity mechanisms.

## Introduction

Activity-driven plasticity in cortical neurons is essential for brain function. Our incomplete understanding of brain diseases highlights fundamental knowledge gaps regarding activity-induced changes in the brain. Classical studies identified activity-induced plasticity genes following seizure (Nedivi et al., 1993; Loebrich and Nedivi, 2009), while recent analysis of cell type-specific activity-regulated transcriptional programs revealed the breadth of activity-dependent mechanisms (Hrvatin et al., 2018). Studies also identified proteomic changes following increased brain activity (do Canto et al., 2020); however, identification and characterization of activity-regulated newly synthesized proteins (NSPs) from identified neural cell types remains an important outstanding question. We addressed this topic by conducting an unbiased screen of differentially expressed NSPs in cortical glutamatergic neurons following pentylenetetrazol (PTZ)-induced seizure.

Recent mass spectrometry-based proteomic studies identified activity-induced NSPs (Alvarez-Castelao et al., 2017; Evans et al., 2020), including novel candidate plasticity proteins induced in response to visual experience (Liu et al., 2018), indicating that proteomic approaches can reveal additional activity-responsive proteins and signaling pathways, commensurate with the complexity of the proteomic landscape (Sharma et al., 2015; Fingleton et al., 2021). Labeling NSPs through the incorporation of azide-containing noncanonical amino acid (ncAA) methionine analogs combined with click chemistry conjugation to alkynes, also known as bio-orthogonal ncAA tagging (BONCAT; Dieterich et al., 2006), has been an important approach to study protein dynamics (Howden et al., 2013; Saleh et al., 2019). The introduction of biotinylated peptide enrichment methods allowed efficient recovery together with direct detection of biotin-modified peptides, called DiDBiT (Direct Detection of Biotin-containing Tags; Schiapparelli et al., 2014), improved BONCAT for identification and quantification of nascent proteomes *in vivo* (McClatchy et al., 2015; Liu et al., 2018; Shah et al., 2022a,b). These studies used native translational machinery to incorporate the ncAA azidohomoalanine (AHA) into NSPs in all cell types. A critical advance restricted ncAA incorporation into genetically targeted cells (Mahdavi et al., 2016; Krogager et al., 2018), for instance, by expression of a mutant methionine tRNA synthetase (mMetRS) that only charges the methionine analog, azidonorleucine (ANL), which cannot be incorporated into proteins by endogenous translational machinery (Ngo et al., 2009; Mahdavi et al., 2016). These strategies enable the analysis of cell type-specific NSPs within intact animals (Alvarez-Castelao et al., 2017; Krogager et al., 2018); however, labeling protocols used in these studies extended over several weeks, possibly resulting in cumulative labeling of “baseline” NSPs before exposure to plasticity-inducing stimuli, obscuring detection of plasticity-induced NSPs.

We sought to quantify cell type changes in activity-induced NSPs. We expressed mMetRS in cortical glutamatergic neurons using EMX-cre and identified ANL-labeled NSPs within 20 h after PTZ-induced seizure in adult mice. First, we provided evidence that mMetRS expression is not detrimental to animal development or behavior. Then, we incorporated several improvements to NSP labeling and analysis protocols. We optimized temporal control of ANL delivery in the brain, guiding our experimental design for PTZ and ANL treatments, and tissue collection. We combined DiDBiT with tagging PTZ and control samples with heavy and light biotin-alkynes, improving detection and quantitation of NSPs. We compared baseline AHA-labeled NSPs with ANL-labeled NSPs from mMetRS-expressing mice. AHA-labeled NSPs included proteins from diverse brain cell types, whereas ANL-labeled NSPs were largely from excitatory neurons. PTZ significantly affected ∼300 NSPs related to synaptogenesis, cytoskeletal dynamics, GTPases, and G-protein-coupled receptors (GPCRs). PTZ also increased nuclear NSPs, including Rad21 and SMARCA2, which regulate chromatin remodeling. The depth of our dataset permitted interrogation of ChipSeq databases to identify upstream regulators of NSPs, providing both retrospective and prospective information regarding activity-dependent proteomic modifications in cortical excitatory neurons in intact animals. Together, our unique experimental achieved improved spatial and temporal labeling of activity-induced NSPs with cell-type resolution combined with enrichment and direct tandem mass spectrometry (MS/MS) detection of biotin-tagged peptides and an improved quantitative strategy using heavy and light tags. By combining the DiDBiT pipeline and sample multiplexing with heavy and light isotopic tags, we enhanced increased signal-to-noise in our proteomic datasets, and consequently improved quantitative analysis of activity-induced changes across animals and conditions.

## Materials and Methods

### Experimental model

All animal experiments were conducted in accordance with the guidelines of the Institutional Animal Care and Use Committee at the Scripps Research Institute (Protocol #08–0082 and #08–0083).

*EMX1^cre^* and *STOPflox R26-MetRS* (C57BL/6-Gt(ROSA)26Sor^tm1(CAG-GFP,-Mars*L274G)Esm^/J mice (stock #028071, The Jackson Laboratory) were crossed to create conditional lines. All the animals are housed and analyzed based on protocols approved by the Institutional Animal Care and Use Committee at the Scripps Research Institute. Both female and male mice were included in the studies.

### l-ANL administration and PTZ-induced seizure

Adult mice received intraperitoneal (i.p.) injections of 830 mg/kg ANL (Ne-azido-L-lysine hydrochloride; CCT, catalog #30456-1G; 400 mm in double-distilled H_2_O, adjusted to pH 7 with NaOH; 10 ml/kg volume-to-body weight ratio) for either 1 or 7 d.

Mice received one i.p. injection of either 47 µg/kg PTZ or saline 30 min after the ANL injection for animals treated with ANL for 1 d. For mice treated for 7 d with ANL, 30 µg/kg PTZ was delivered 30 min after the last ANL injection. Animals were monitored for 20 min after the PTZ injection, during which seizure behavior was scored with the Racine seizure scale. If 30 µg/kg PTZ treatment did not induce seizure, an additional 8–9 µg/kg was delivered. Animals that had seizure behavior stronger or as strong as a generalized tonic-clonic seizure were selected for proteomic analysis. Brain tissue was harvested and frozen in cold isopentane 18–20 h after the PTZ treatment.

### Free l-ANL detection in brain tissue by LC-MS

To measure pharmacokinetics of ANL in brain tissue, ANL (400 mm, dissolved in water and adjusted to pH 7 with 10N NaOH) was administered to a cohort of adult female and male mice (3–4 mice/group; age, 3–5 months; body weight is within 25–30 g for females and 28–33 g for males) through injection at a dosage of 10 ml/kg, i.p. Brains were acutely extracted and snap frozen in isopentane on dry ice at 0.5, 1.5, 2, 4, 8, 12, 18, and 24 h after injection (*N* = 3 mice were included per time point). Brain samples, consisting of 10–20 mg of tissue, were homogenized in 4:1 MeOH/0.1% formic acid. Extracts were harvested, evaporated to dryness in a SpeedVac, and reconstituted in 0.1% formic acid. AHA was spiked in as internal standard. Samples were then analyzed by liquid chromatography (LC)-MS. LC conditions were set as follows: column = Ethylene BridgedHybrid (BEH) amide (Waters), 2.1 × 100 mm; flow rate = 200 µl/min; MPA (mobile phase A) = 50 mm NH_4_OAc; MPB (mobile phase B) = ACN (Acetonitrile); gradient: T0 = 5:95; T0.1 = 5:95; T5 = 50:50; T8 = 50:50; T8.1 = 5:95; T16 = off. The transition states monitored were as follows:

(1) L−Azidohomoalanine:144.78 −>70.99 cone voltage=6V, collision energy=6V,

(2) L−Azidonorleucine:172.94 −>70.14 cone voltage=6V, collision energy=12V172.94 −>82.05 cone voltage=6V, collisionenergy=8V.

### ANL treatment in HEK293T cells

HEK cells were cultivated in a 150 cm^3^ flask to reach 80% confluence in DMEM media containing 10% FBS. Cells were washed in Dulbecco's PBS three times, and media were replaced with DMEM methionine (–), 5% dialyzed FBS, and 4 mm ANL. Cells were incubated in these media for 48 h, washed, pelleted in 15 ml Falcon tubes, and frozen until use. Experiments were performed in triplicate.

### Immunohistochemistry in brain sections

Mice were anesthetized with isoflurane and fixed with 4% paraformaldehyde (PFA)/PBS by intracardiac perfusion. The brains were then harvested and postfixed with 0.4% PFA/PBS overnight at 4°C, and 90-µm-thick coronal vibratome sections were cut in cold PBS. Free-floating sections from bregma −1.4 to −2.5 mm were taken and briefly boiled in antigen retrieval buffer (100 mm sodium citrate, 0.05% Tween-20 in PBS, with pH adjusted to 6.0). Sections were then washed three times in room temperature PBS, blocked in buffer (4% bovine serum albumin, 3% normal donkey serum, 0.1% Triton X-100, and 0.5% sodium azide in PBS) at room temperature for 1 h, and incubated overnight at 4°C with the following primary antibodies diluted in blocking solution: 1:200 mouse anti-NeuN (catalog #MAB377, Millipore; RRID:AB_2298772; 1:500 chicken anti-GFP (catalog #GFP-1010, Aves Labs; RRID:AB_2307313; 1:250 rabbit anti-Cux2 (catalog #sc-13 024, Santa Cruz Biotechnology; RRID:AB_2261231); 1:500 rat anti-Ctip2 (catalog #ab18465, Abcam; RRID:AB_2064130); 1:1000 mouse anti-Gad67 (catalog #MAB5406, Millipore; RRID:AB_2278725); and 1:1000 mouse anti-GFAP (catalog #MAB360, Millipore; RRID:AB_11212597). The following day, the sections were washed three times with PBS, incubated for 3 h in a 1:200 dilution of the following secondary antibodies (anti-goat Alexa Fluor 488; catalog #A11055; RRID:AB_2534102; and anti-mouse Alexa Fluor 564; catalog #A11004; RRID: AB_2534072; Thermo Fisher Scientific), then washed five times with PBS and mounted on glass slides with PVA-DABCO (catalog #10981, Sigma-Aldrich).

### Acquisition and analysis of confocal images

Brain regions of interest were identified using the Allen Brain Atlas (http://mouse.brain-map.org/static/atlas). Fluorescent images were acquired with a confocal microscope (model C2, Nikon) with a 20× objective. Camera settings were adjusted for each channel and applied to all images in the same dataset. Conventional image analysis was conducted using Nikon Elements and FIJI software packages, where the refining processing is applied to all pixels.

### Behavior

#### Open field.

Locomotor activity and anxiety level of the animal were assessed using an open field test. Animals were acclimated to the behavior room for 30 min before the test and allowed to move freely for 30 min in polycarbonate cages (42 × 22 × 20 cm) placed in frames (25.5 × 47 cm) mounted with two levels of photocell beams positioned 2 and 7 cm above the bottom of the cage (San Diego Instruments), as we described previously (Zhu et al., 2019). Activity Monitor 7 (Product #SOF-812, Med Associates) software was used to monitor and analyze the signal acquired from the photocell beams 6 min after the initial deposition. Multiple activity parameters were analyzed, including walking distance, ambulatory counts, and vertical counts indicating the locomotor activity of the animals. The arena was divided into central and residual areas. The time an animal spent in each area was compared to indicate their anxiety level.

#### Contextual fear conditioning.

The associative memory of the animal was examined using contextual fear conditioning (CFC). Mice were acclimated to the behavior room for 30 min before the test, then were introduced to the fear-conditioning boxes (Med Associates) with specific visual and odor contexts (wall decorated with picture of patterned geometric features and olfactory stimulus from a 0.1% acetic acid solution). After 3 min, animals were subjected to four short electric footshocks of 0.55 mA amplitude with 1 min intershock intervals. Memory tests were performed 2 or 24 h after training with 3 min exposure to the same training box. Freezing was determined in 0.75 s bouts, and the freezing percentage is calculated according to the time spent in the context, as we described previously (Zhu et al., 2019).

### Experimental design and statistical analysis

**Figure 1.**
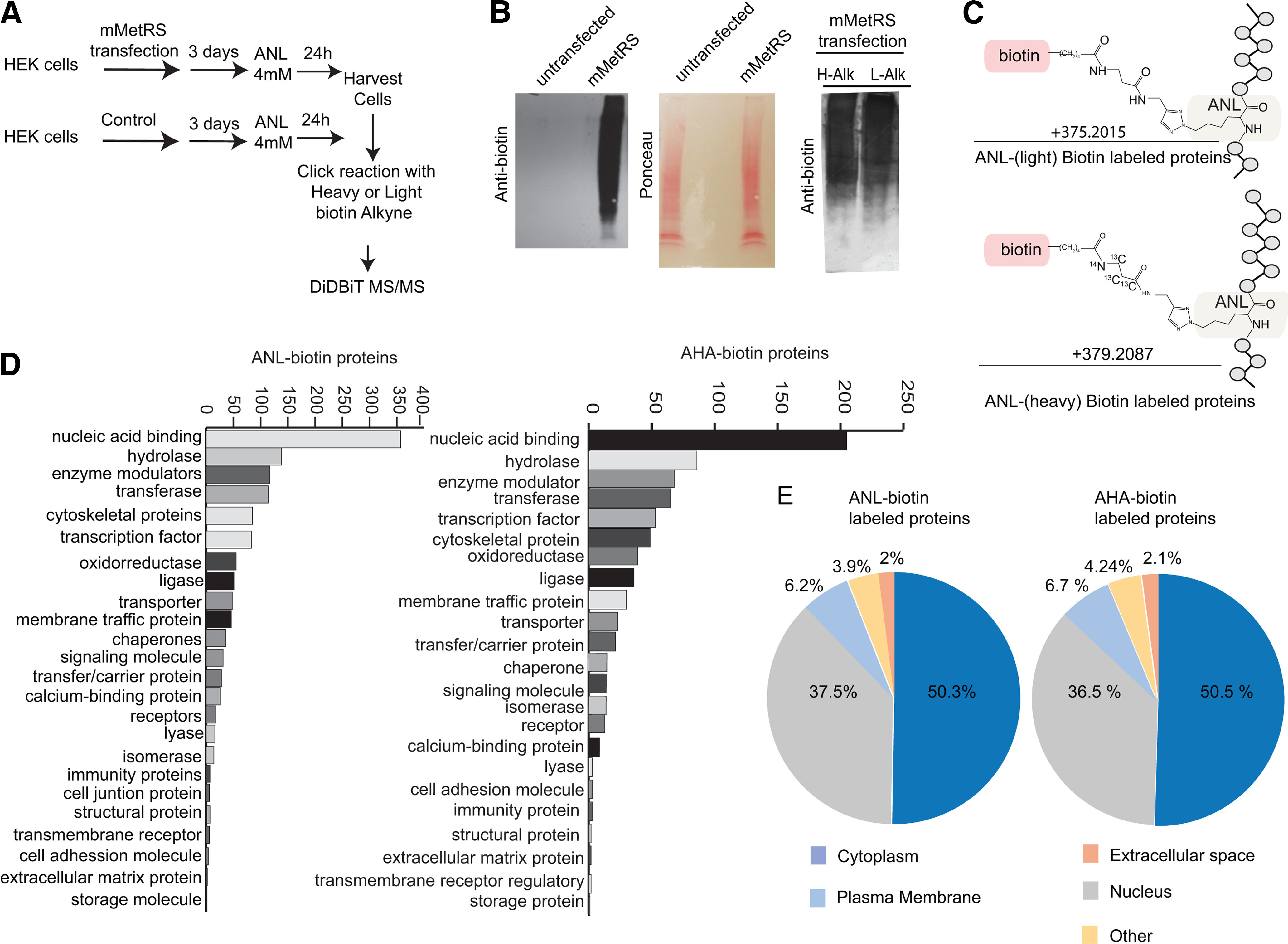
Direct detection of newly synthetized ANL-biotin-labeled proteins in mMetRS expressing HEK293T cells. ***A***, Protocol for *in vitro* ANL labeling. HEK cells were transfected using lipofection with mMetRS and incubated with 4 mm ANL in methionine-free media for 24 h. Cell pellets were washed, homogenized, and processed for click chemistry with heavy or light biotin alkyne. After click reactions, proteins were precipitated and digested, and ANL-biotin-tagged peptides were isolated on NeutrAvidin using the DiDBiT protocol. ***B***, Left, mMetRS-transfected cells, but not untransfected cells, showed incorporation of ANL. Western blots for biotin (left) and corresponding Ponceau staining (right). Right, Western blots of ANL-biotin-tagged proteins from click reactions performed with heavy or light biotin-alkyne show comparable biotin labeling. H-Alk, heavy-alkyne; L-Alk, light-alkyne. ***C***, Chemical structure of the ANL-biotin modification on methionine sites in peptides, with +375.2015 and +379.2087 mass gain for light and heavy biotin-alkynes, respectively, used for direct mass spectrometry identification of tagged peptides. ***D***, Comparable representation of protein classes in ANL-labeled and AHA-labeled newly synthesized proteins using Panther (Extended Data Fig. 1-1, see for proteins in the different categories). ***E***, Comparable subcellular distribution of newly synthesized proteins labeled by ANL and AHA incorporation, according to Ingenuity Pathway Analysis (Extended Data Fig. 1-1, lists of proteins).

**Figure 2.**
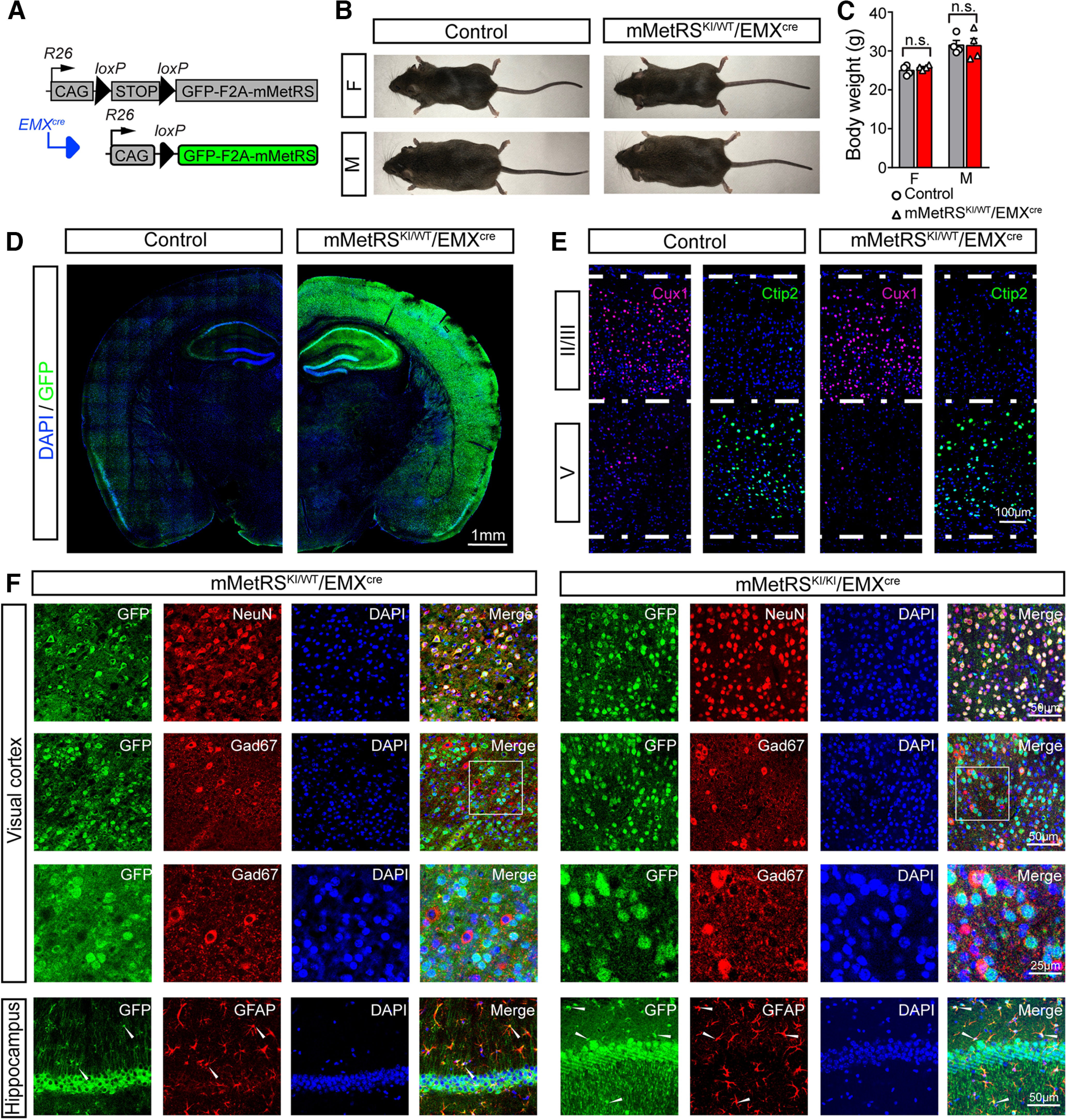
Characterization of mMetRS/EMX^cre^ lines. ***A***, Schematic of the breeding strategy. EMX^cre^ drives expression of GFP-F2A-mMetRS from the Rosa26floxSTOP-GFP-F2A-mMetRS allele, allowing GFP expression to report mMetRS distribution. ***B***, Images of age-matched animals of mMetRS/EMX^cre^ mutants and their MetRS-negative control littermates at postnatal day 60. ***C***, Body weights of male and female mMetRS/EMX^cre^ mutants and control littermates are comparable (individual datapoints and mean ± SEM values; *N* = 4, paired *t* test with Welsh's correction, n.s., not significant). ***D***, GFP expression in cortex and hippocampus in coronal brain sections counterstained with DAPI. ***E***, Confocal images of immunolabeling of layer-specific excitatory cortical neurons with antibodies to Cux1 and Ctip2 in layers II/III and V in DAPI-stained coronal sections in visual cortex, indicating that mMetRS expression does not affect the development of cortical lamination. ***F***, Cellular specificity of GFP-F2A-mMetRS expression in mMetRS^KI/WT^/EMX^cre^ and mMetRS^KI/KI^/EMX^cre^ mice. Coronal sections through visual cortex and hippocampus were labeled with DAPI and antibodies against markers of all neurons (NeuN), inhibitory GABAergic neurons (GAD67), or astrocytes (GFAP). GFP is present in NeuN^+^ neurons, but not in GABAergic neurons. Some GFAP^+^ astrocytes express GFP, which is consistent with the known EMX expression pattern.

**Figure 3.**
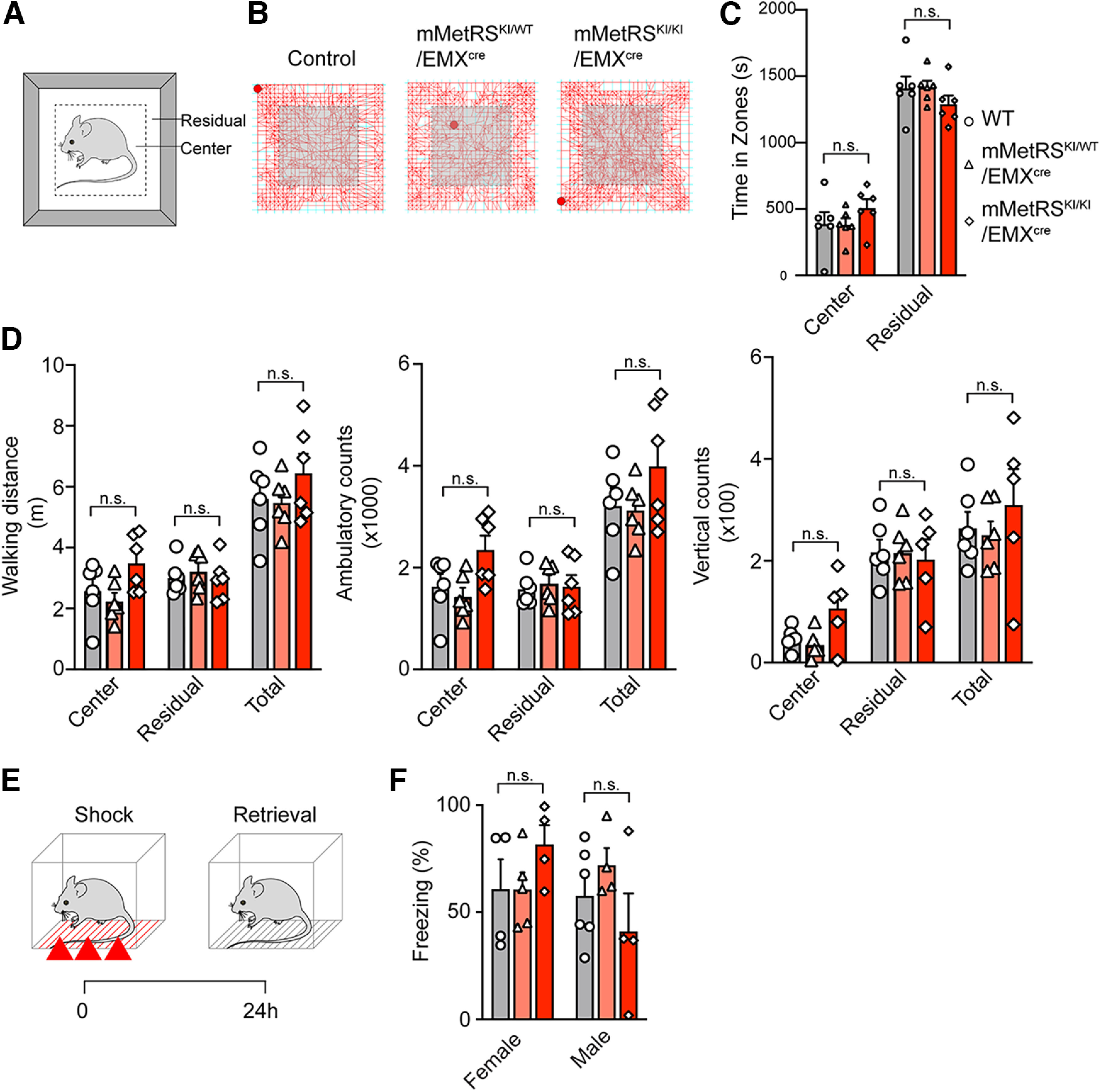
mMetRS expression in excitatory cortical neurons does not affect behavior. ***A–D***, Open field tests of locomotor activity. ***A***, Schematic of the open field arena, showing the center and peripheral (residual) regions of the arena. ***B***, Representative foot tracks of mMetRS^KI/WT^/EMX^Cre^ mutants (middle) and mMetRS^KI/KI^/EMX^Cre^ mutants (right), and their control littermates (left). ***C***, Quantification of time spent in central and peripheral zones. ***D***, Quantification of cumulative walking distances, ambulatory counts, and vertical counts. ***E***, ***F***, Analysis of associative memory. ***E***, Workflow for fear conditioning and contextual memory retrieval. Animals are exposed to a series of 3 footshocks and tested for memory retrieval assessed as freezing behavior in the same context 24 h later. ***F***, Short- and long-term memory in males and females, assessed as a percentage of the time spent freezing. All graphs show individual datapoints and averaged values (mean ± SEM). ***A–D***, *N* = 6 (3 males and 3 females). ***F***, *N* = 4-6, paired *t* test with Welsh's correction. ***C***, ***D***, ***F***. Gray, WT; pink, mMetRS^KI/WT^; red, mMetRS^KI/KI^. n.s., not significant.

Details of experimental designs are provided in Results, figures, and figure legends. Sample sizes were selected based on preliminary experiments and our published studies. For the data shown (see Figs. 2, 3), means and SEMs were calculated in Prism GraphPad, and statistical significance was calculated using Student's *t* test with Welch's correction, taking into consideration normality of the data distribution. Similarly, Western blot data were analyzed with a paired *t* test between pairs of PTZ and control replicates in Prism. *N* values, numbers of males and females analyzed, and statistical tests are provided in the figure legends. We run quality controls on the protein samples before running the MS/MS to ensure that the click chemistry and peptide isolation steps meet standards required to proceed with the MS/MS. The experiments used in the analysis in Figure 1 (also see Figs. 5, 6, 7) met these standards, and we identified similar numbers of proteins by MS/MS across samples that were compared. The analysis (see Fig. 7*E*) suggests that the effect of PTZ on the representation of protein components within signaling pathways varies across samples. Samples run together on the MS/MS instrument tend to have less variance than samples run in separate batches. Samples 1-4 (S1-S4) were run together, and samples 5 and 6 (S5, S6) were run together in a separate batch, so it does not appear that run-to-run differences account for the variance across samples. S3, S5 and S6 were from females, and S1, S2 and S4 were from males. Although the study is not powered to evaluate sex as a variable, it looks like variance within each sex may be comparable. Proteomic quantification and statistical analysis were performed with Integrated Proteomics Pipeline–IP2 (Bruker Scientific, http://www.bruker.com). The statistical significance of the differential expression of all proteins was assessed using a two-tailed paired *t* test of their corresponding peptide quantification ratios between both conditions with *p* < 0.05 being the cutoff for considering statistical significance. False discovery rate (FDR)-adjusted *p* values are calculated using the Benjamini–Hochberg correction. MS data are deposited to ProteomExchange.

### Sample preparation for MS/MS identification of AHA- and ANL-biotin-labeled newly synthesized proteins

#### Protein extract preparation.

Frozen brain cortices or cell pellets (for cell culture) were lysed in 0.5% SDS in PBS plus a cocktail of endogenous protease inhibitors (Complete Protease Inhibitor Cocktail Tablets, Roche) by homogenizing and sonicating with 10 pulses using a tip sonicator (Sonic Dismembrator model 100, Thermo Fisher Scientific). Samples were then boiled for 10 min and cooled to room temperature. Any remaining insoluble material was resuspended with additional sonication pulses. We measured protein concentration using a protein assay kit (BIO-RAD), and aliquots of 0.5 mg of protein suspension were transferred to Eppendorf tubes.

The ANL that was incorporated into proteins was labeled with biotin-alkyne by click chemistry reaction performed in the total protein suspension, as we previously published (Schiapparelli et al., 2014). For quantitative MS analysis, click reactions were performed using biotin-alkyne labeled with heavy stable C^13^ and N^15^ isotopes: biotin-β-alanine-13C3,15N-alkyne (biotin propargyl amide) or the light isotope form of the alkyne (Setareh Biotech) in the different experimental groups (McClatchy et al., 2015). We used 12 mg of protein lysate from brain or cell samples per experimental condition. For each click reaction, we used an aliquot of 0.5 mg of protein lysate and brought the reaction volume to 346 µl with PBS before adding the click reaction reagents. We added the following reagents in sequence, vigorously vortexing after each addition: 30 µl of 1.7 mm tris[(1-benzyl-1H-1,2,3-triazol-4-yl)methyl]amine (TBTA) dissolved in 4:1 tert-butanol/DMSO, 8 µl of 50 mm CuSO4 dissolved in ultrapure water, 8 µl of 5 mm biotin-alkyne (in light or heavy form) dissolved in DMSO, and 8 µl of 50 mm TCEP dissolved in water. The click reactions were vortexed and incubated at room temperature for 1–2 h or overnight with gentle rotation at 4°C.

We loaded 20 µl of each click reaction in Western blot gels for analyzing biotin-alkyne incorporation to proteins transferring to nitrocellulose membranes and incubating with anti-biotin antibody, as described above. The click reactions of both experimental groups were mixed 1:1, vortexed, and proteins are precipitated with methanol/chloroform. Pellets containing biotinylated proteins were air dried for 10 min.

#### Protein digestion.

HEK cell protein precipitates were resuspended by adding 200 µl of 8 m urea and 200 µl of 0.2% ProteaseMAX surfactant (Promega) dissolved in 50 mm NH4HCO3. The protein suspension was reduced by adding tris(2-carboxyethyl)phosphine (TCEP; Sigma-Aldrich) to 5 mm final concentration and incubated at 55°C with vigorous orbital shaking using a Thermomixer (Eppendorf). Protein alkylation was done by adding iodoacetamide (Sigma-Aldrich) to a 10 mm final concentration and incubating with vigorous rotation in the dark for 20 min. To digest the proteins, we added the following in order: 150 µl of 50 mm NH4HCO3, 2.5 µl of 1% ProteaseMAX dissolved in 50 mm NH4HCO3, and 1:100 (enzyme/protein, w/w) sequencing (seq) grade trypsin (Promega) to a final reaction volume of 500 µl. The digestion reactions were incubated for 3.5 h at 37°C with vigorous orbital shaking using a Thermomixer (Eppendorf).

For mouse brain cortex samples, TrypZean was used for protein digestion as previously published (McClatchy et al., 2020). Briefly, protein pellets were resuspended in an 8 m urea, 100 mm Tris HCl, pH 8, buffer with sonication. Proteins were then reduced and alkylated as mentioned above and diluted in 100 mm Tris HCl buffer to dilute urea to 2 m. TrypZean (Sigma-Aldrich) was added 1:25 (enzyme/protein, w/w), and samples were incubated at 37°C overnight with vigorous shaking.

#### Biotinylated peptide enrichment for mass spectrometry identification.

Samples were centrifuged at 20,000 × *g* for 20 min at room temperature to remove undigested insoluble material and supernatant containing the peptide mixture was collected in an Eppendorf tube. Any remaining peptides in the insoluble pellet were extracted by adding 0.5 ml of 0.1% trifluoroacetic acid (TFA) in water, resuspending the pellet by pipetting and centrifuging again for 20 min. The supernatant was pooled with the previous one before desalting using Sep-Pak tC18 solid-phase extraction cartridges (Waters). Before loading the mixture of peptides, the cartridges were washed sequentially with 3 ml of acetonitrile, 3 ml of 0.5% acetic acid, 50% acetonitrile in water, and 3 ml of 0.1% TFA in water. After loading the peptide mixtures, the cartridges were washed with 3 ml of 0.1% TFA and then with 0.250 ml of 0.5% acetic acid in water. The peptides were eluted into a clean tube with 1 ml of 0.5% acetic acid, 80% acetonitrile in water, and dried in Eppendorf tubes in a Speed Vac (Thermo Fisher Scientific). The dried peptide pellets were solubilized in 1 ml of PBS and incubated with a 150 µl slurry of NeutrAvidin beads (Thermo Fisher Scientific) in a rotator overnight at 4°C. The beads were precipitated by centrifugation at 1000 × *g* for 5 min, and the flow through of unbound peptides was collected for MS analysis. The beads were washed three times in 5% acetonitrile in PBS, three times in PBS alone, and three times with ultrapure water. Excess liquid was completely removed from the beads using a micropipette, and biotinylated peptides were eluted by adding 0.3 ml of solution containing 0.2% TFA, 0.1% formic acid, and 80% acetonitrile in water. The beads were centrifuged at 1000 × *g*, and the first elution of biotinylated peptides was transferred to an Eppendorf tube. A second elution of 0.3 ml was incubated at 70°C with vigorous agitation for 5 min for maximum release of peptides from the beads. The two peptide elutions were pooled and used for MS identification.

### Protein identification by LC-MS/MS

The resulting peptides from the HEK cells and AHA-labeled cortices were pressure loaded onto a 250 µm inner diameter (i.d.) capillary with a Kasil Frit containing 2 cm of 10 µm Jupiter C18-A material (Phenomenex) followed by a 2 cm of 5 µm Partisphere strong cation exchanger (Whatman). This column was washed with Buffer A (95% water, 5% acetonitrile, 0.1% formic acid) after loading. A 100 µm i.d. capillary with a 5 µm pulled tip packed with 15 cm of 4 µm Jupiter C_18_ material (Phenomenex) was attached to the loading column with a union, and the entire split-column (loading column–union–analytical column) was placed in line with an Agilent 1100 quaternary HPLC. The DiDBiT samples were analyzed using a modified 5- or 10-step MudPIT separation described previously (Schiapparelli et al., 2019). As peptides eluted from the microcapillary column, they were electrosprayed directly into a mass spectrometer (model Elite, Thermo Fisher Scientific) with the application of a distal 2.4 kV spray voltage. A cycle of one full-scan FT mass spectrum [300–1600 mass-to-charge ratio (*m*/*z*)] at 240,000 resolution, followed by 20 data-dependent IT MS/MS spectra at a 35% normalized collision energy, was repeated continuously throughout each step of the multidimensional separation.

The resulting peptides from the ANL brain samples were resuspended in 30 µl of Buffer A. Ten microliters of the peptide suspension were loaded on a column self-packed with BEH (Waters; inner diameter, 100 µm × 1.7 µm × 20 mm) and eluted using a 1–30% gradient of solvent B for 160 min, 30–90% for 60 min, and 90% for 20 min at a 200 µl/min flow rate using an Easy-1000 UPLC coupled to an Orbitrap Lumos Tribid (Thermo Fisher Scientific). This analysis was performed twice for each biological replicate. The MS1 spectra were recorded in the Orbitrap with *R* = 120,000 with a mass range of 400–1,500 *m/z* and an automatic gain control (AGC) of 4 × 10^5^ counts. The cycle time was set to 3 s, and within these 3 s, the most abundant ions per scan were selected for CID MS/MS in the ion trap with an AGC target of 2 × 10^4^ ions and minimum intensity of 5,000. Maximum fill times were set to 50 and 35 ms for MS and MS/MS scans, respectively. Quadrupole isolation at 1.6 m/z was used, monoisotopic precursor selection was enabled, charge states of 2–7 were selected, and dynamic exclusion was used with an exclusion duration of 5 s. For all analyses, application of the mass spectrometer scan functions using data-dependent acquisition and HPLC solvent gradients were controlled by the Xcalibur data acquisition software.

### Processing mass spectra and protein identification

For ANL data, MS/MS spectra were searched with the ProLuCID algorithm (Xu et al., 2015) against the UniProt mouse database (release date, April 17, 2017) concatenated to a decoy database in which the sequence for each entry in the original database was reversed. The following modifications were searched for a static modification of 57.02146 on cysteine for all analyses, a differential modification on methionine of 379.2087 (heavy) and 375.2015 (light) for using the Setareh Biotech biotin-alkynes. For the AHA data, a differential modification of 452.2376 on methionine was searched. ProLuCID algorithm results were assembled and filtered using the DTASelect program (version 2.0) (Lavallée-Adam et al., 2015). DTASelect 2.0 uses a linear discriminant analysis to dynamically set XCorr and DeltaCN thresholds for the entire dataset to achieve a user-specified FDR. In addition, the modified peptides were required to be partially tryptic, a <20 ppm deviation from peptide match, and an FDR at the spectra level of 0.01. The FDRs are estimated by the program from the number and quality of spectral matches to the decoy database. For all datasets, the protein FDR was <1% and the peptide FDR was <0.5%.

The algorithm Census was used to quantitate the differential abundances between the light and heavy biotin alkyne-ANL-labeled peptides (Park et al., 2014).

### Western blots

Protein extracts were prepared as described above. After click reaction with biotin-alkyne, proteins were quantified as mentioned above and solubilized by diluting 1:1 in a buffer containing 8 m urea and 1% SDS, sonicating using the tip sonicator, and centrifuging at 10,000 × *g* for 10 min at room temperature. Supernatants (inputs) were incubated overnight at 4°C with 20 µl of high-capacity NeutrAvidin beads (Pierce NeutrAvidin Agarose; catalog #29201, Thermo Fisher Scientific) previously washed in PBS. After incubation, the beads were pelleted by centrifuging for 5 min at 1000 × *g* for separating supernatant (flow through) and later were washed four times with 4 m urea and 1% SDS buffer. The biotinylated proteins were eluted from the beads by boiling in 40 µl of Laemmli sample buffer (catalog #1610737, BIO-RAD) plus 2% β-mercaptoethanol.

Inputs and NeutrAvidin purified protein were run in a Mini-PROTEAN electrophoresis cell (catalog #1658004, BIO-RAD; and compatible precast gels) and transferred to nitrocellulose membranes using a Trans-Blot Turbo Transfer System (catalog #1704150, BIO-RAD; and compatible transfer kits). The membranes were blocked with 5% blocking reagent (catalog #1706404, BIO-RAD) in TBST (136 mm NaCl, 20 mm Tris, pH adjusted to 7.0, 0.05% Tween 20) for 1 h at room temperature, incubated overnight at 4°C with the following primary antibodies in blocking buffer: polyclonal goat anti-biotin antibody (1:1000; catalog #31852, Thermo Fisher Scientific; RRID:AB_2096845); rabbit monoclonal anti-Smarca2 (1:1000; catalog #ab240648, Abcam); mouse monoclonal anti-Rad21 (1:1000; catalog #NB100-386, Novus Biologicals; RRID:AB_10000864); rabbit polyclonal anti-ARHGEF2 (1:1000; catalog #ab155785, Abcam; RRID:AB_2818944); rabbit monoclonal anti-NCAM (1:1000; catalog #NBP2-66 968, Novus Biologicals); and mouse monoclonal anti-ANHAK (1:1000; catalog #ab68556, Abcam; RRID:AB_1209218). Membranes were then washed with TBST three times for 10 min and incubated in blocking buffer with the following secondary antibodies: rabbit-anti-goat conjugated with HRP (1:2500; catalog #172–1034, BIO-RAD; RRID:AB_11125144); goat anti-rabbit conjugated with HRP (1:2500; catalog #172–1019, BIO-RAD; RRID:AB_11125143; and goat anti-mouse conjugate with HRP (1:2500; catalog #172–1011, BIO-RAD; RRID:AB_11125936) for 2 h at room temperature. Membranes were washed again in TBST, incubated with Pierce ECL (catalog #32106, Thermo Fisher Scientific) or SuperSignal West Fempto (catalog #34095, Thermo Fisher Scientific), and developed with x-ray film in a darkroom. The signal of the NeutrAvidin-enriched proteins were normalized over the signal of the inputs. Signal quantification is performed with FIJI (ImageJ) software.

## Results

### Mutant MetRS expression allows genetic control of analysis of newly synthesized proteins

To characterize the use of mMetRS and ANL incorporation into newly synthesized proteins and to compare the use of mMetRS/ANL with AHA labeling in BONCAT, we first used HEK cells transfected with mMetRS and incubated cultures with 4 mm ANL in methionine-free media for 24 h. Proteins were processed for click chemistry to conjugate ANL with heavy or light biotin-alkyne, digested with protease, and biotinylated peptides were purified by DiDBiT for mass spectrometry analysis (Fig. 1*A*). Western blots with anti-biotin antibody show comparable levels of ANL incorporation and biotin-alkyne labeling with heavy and light biotin-alkynes, whereas no ANL incorporation was detected in untransfected HEK cells (Fig. 1*B*). Heavy and light versions of biotin-alkyne produce distinct mass shifts, permitting MS detection and quantification of biotin-ANL tags (Fig. 1*C*). We detected 3521 ANL-biotin-modified peptides corresponding to 1932 proteins (Extended Data Fig. 1-1). We compared the ANL-biotin-labeled protein dataset to our previously published dataset of AHA-biotin-labeled newly synthetized proteins in HEK cells (Schiapparelli et al., 2014). Analysis of protein class with the Panther Classification System (Mi et al., 2019) showed that ANL incorporates into similar protein classes in mMetRS-expressing HEK cells as seen with AHA incorporation in untransfected HEK cells and that the most represented proteins are similarly ranked in AHA- and ANL-labeled proteins (Fig. 1*D*). In addition, compartment analysis using Ingenuity Pathway Analysis (IPA; QIAGEN; Krämer et al., 2014) showed that AHA- and ANL-labeled proteins are similarly distributed in the plasma membrane, and cytoplasmic and nuclear compartments (Fig. 1*E*). These results indicate that the combination of direct detection of biotinylated proteins with the mMetRS expression permits the accurate detection and quantification of labeled proteins under the genetic control of ncAA incorporation into newly synthetized proteins, and that AHA- and mMetRS-mediated ANL incorporation identified similar classes of proteins.

10.1523/JNEUROSCI.0707-22.2022.f1-1 Figure 1-1 Data for Figure 1. AHA versus ANL in HEK cells. Download Figure 1-1, XLSX file.

### Characterization of mMetRS^KI^/EMX^cre^ mice as a tool to study cell-specific proteomics

We are interested in evaluating activity-regulated NSPs in neocortical pyramidal neurons using the EMX^cre^ line to drive mMetRS expression. The EMX1 promotor drives expression of cre recombinase in pyramidal neuronal progenitor cells and progeny (Gorski et al., 2002), offering flexibility over an extended time frame for analysis of pyramidal neuron proteostasis. First, we explored whether expressing mMetRS in the brain from mid-embryonic stages affects normal growth and brain function in intact animals. We crossed GFP-2A-mMetRS animals to mice expressing cre recombinase from the EMX1 promotor (Fig. 2*A*). Heterozygous mMetRS animals (mMetRS^KI/WT^) were born at the expected Mendelian ratio and lived a normal life span. They appear normal (Fig. 2*B*) and acquired similar adult body weight compared with their wild-type littermates for both sexes (Fig. 2*C*).

To assess whether brain structure was affected by EMX^cre^-driven mMetRS expression in cortex and hippocampus from E10 to adulthood, we compared immunohistochemical labeling between wild-type mice and both heterozygous and homozygous mMetRS mice. With GFP immunolabeling, we confirmed that cre-dependent GFP expression was only detected in mutant animals and was restricted to neocortex and hippocampus (Fig. 2*D*). Neocortical lamination is critical for specialized laminar functions and the organized directionality of information processing. To assess the integrity of cortical lamination, we labeled coronal sections with layer-specific markers Cux1 (layer II/III) and Ctip2 (layer V) and detected clear laminar boundaries in both mutant and wild-type animals (Fig. 2*E*), suggesting that early-onset mMetRS expression in mouse brain does not affect cortical anatomy.

The EMX^cre^ line expresses cre recombinase in pyramidal neuronal progenitor cells and progeny resulting in cre recombinase expression in excitatory pyramidal neurons and some astrocytes (Gorski et al., 2002). The STOPflox R26-mMetRS line has been shown to recombine strictly following the cell type-specific expression of cre recombinase in the CaMKII-Cre and Gad1-Cre lines with which it was crossed (Alvarez-Castelao et al., 2017, 2019). We tested whether the cell type specificity achieved in mMetRS^KI^/EMX^cre^ mice is consistent with the cre recombinase expression pattern in mice that are heterozygous or homozygous for mMetRS. Using NeuN as a pan-neuronal marker, we find that all GFP signals colocalize with NeuN in the hippocampus and visual cortex (Fig. 2*F*). We find no colocalization of the inhibitory neuron marker GAD67 with GFP in cortex (Fig. 2*F*) or hippocampus (data not shown). We find some colocalization between GFAP, an astrocyte marker, and GFP, but GFAP^+^/GFP^+^ cells were a minority compared with NeuN^+^/GFP^+^ cells (Fig. 2*F*, bottom row). These results lay the foundation for our following experiments of cell type-specific nascent proteome identification and analysis.

To assess whether mMetRS expression affects brain function, we evaluated several behaviors in the mMetRS^KI^/EMX^cre^ mice. First, locomotor activity was measured and compared to determine any defect on motor control and arousal state. Animals were introduced to an open arena and allowed to move about freely (Fig. 3*A*), while multiple motion-related parameters were collected. Because mice are light aversive, they tend to avoid brightly lit areas such as the center of the arena, especially when they are under stress. Therefore, the anxiety level of the mice can be evaluated by tracking the time spent in the central and peripheral compartments of the arena. mMetRS^KI/WT^ or mMetRS^KI/KI^ (heterozygotic or homozygous) mice and littermate controls spent comparable amounts of time in both compartments (Fig. 3*B*,*C*), indicating no difference in anxiety level in the mutants and controls. Furthermore, we detected no difference in running distance, mobility, or rearing behavior (Fig. 3*D*), suggesting that mMetRS mice display typical levels of activity, normal hindlimb muscular strength, and healthy motor function.

Since the hippocampus and cortex are well known to be major hubs for learning and memory processing, we evaluated CFC (Fig. 3*E*) to test whether mMetRS mutants have impaired associative memory. By comparing the percentage of the time during which they froze in the training box where they were previously footshocked, we found that male and female mutants were able to recognize the training box environment and associate it with previous adverse experience (Fig. 3*F*). Together, these data indicate that heterozygous or homozygous mMetRS expression from early developmental stages does not grossly affect neocortical structure or function.

### Genetic control of mMetRS expression allows identification of NSPs in specific neural cell types

**Figure 4.**
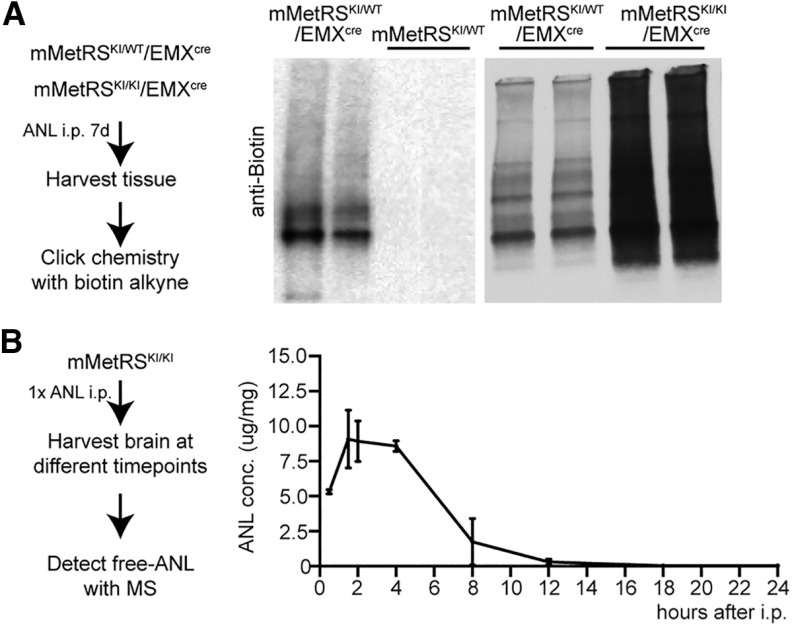
Optimization of ANL protein labeling *in vivo*. ***A***, Comparison of ANL incorporation in heterozygote or homozygote mMetRS^KI/WT^/EMX and mMetRS^KI/KI^/EMX mice and mMetRS/WT littermates. Left, Schematic of the protocol. Animals were treated with a daily dose of ANL (830 mg/kg, i.p.) for 1 week, and the brain was harvested 18–20 h after the last ANL treatment. Tissue was processed for click chemistry with biotin-alkyne. Middle, Western blots comparing ANL-biotin-labeled proteins from mMetRS^KI/WT^/EMX^cre^ and mMetRS^WT/WT^ littermates. No ANL incorporation into proteins is observed in the absence of cre-induced mMetRS expression. Right, Western blots showing more ANL-biotin label in comparable amounts of protein samples from mMetRS^KI/KI^EMX^cre^ mice compared with mMetRS^KI/WT^/EMX^cre^ mice. ***B***, Analysis of brain ANL levels after intraperitoneal injection by mass spectrometry. Left, Schematic of the experiment. mMetRS mice received 1 dose of ANL intraperitoneally and brains were harvested at different timepoints after injection. Free ANL in the brain was detected by MS. Right, ANL levels in the brain over time [mean ± SEM; *N* = 4 mice (2 males and 2 females) paired *t* test with Welsh's correction]. ANL concentration was calculated by the amount of ANL in ug normalized to the amount of brain tissue analyzed in mg.

To evaluate the cell type specificity of labeling newly synthesized proteins in mMetRS^KI^/Emx^cre^ mice, we compared the nascent proteome labeled by incorporation of ANL, which requires the presence of mMetRS, to the nascent proteome labeled by incorporation of AHA, which is incorporated into newly synthesized proteins in all cell types using wild-type MetRS (Dieterich et al., 2006). We administered ANL intraperitoneally daily over 1 week to mMetRS^KI^/Emx^cre^ or mMetRS mice, collected brains, and tagged ANL in proteins with biotin-alkyne using click chemistry. We detected ANL-biotin-labeled proteins in samples from mMetRS^KI^/Emx^cre^ animals but not in littermates by Western blot, confirming the requirement for mMetRS for the incorporation of ANL into proteins (Fig. 4*A*, left and middle). Furthermore, samples from homozygous mMetRS^KI/KI^ mice were more intensely labeled with biotin than samples from heterozygous mMetRS^KI/+^ mice (Fig. 4*A*, right), indicating greater ANL incorporation into mMetRS^KI/KI^ mice. To evaluate the pharmacokinetics of ANL in the brain following intraperitoneal ANL delivery, we determined the free ANL concentration by MS/MS in brain samples collected at different timepoints after a single ANL intraperitoneal injection (Fig. 4*B*, left). ANL concentrations in the brain peak between 2 and 4 h after injection and persist for 8 h. ANL levels are undetectable by 16 h after injection (Fig. 4*B*, right).

**Figure 5.**
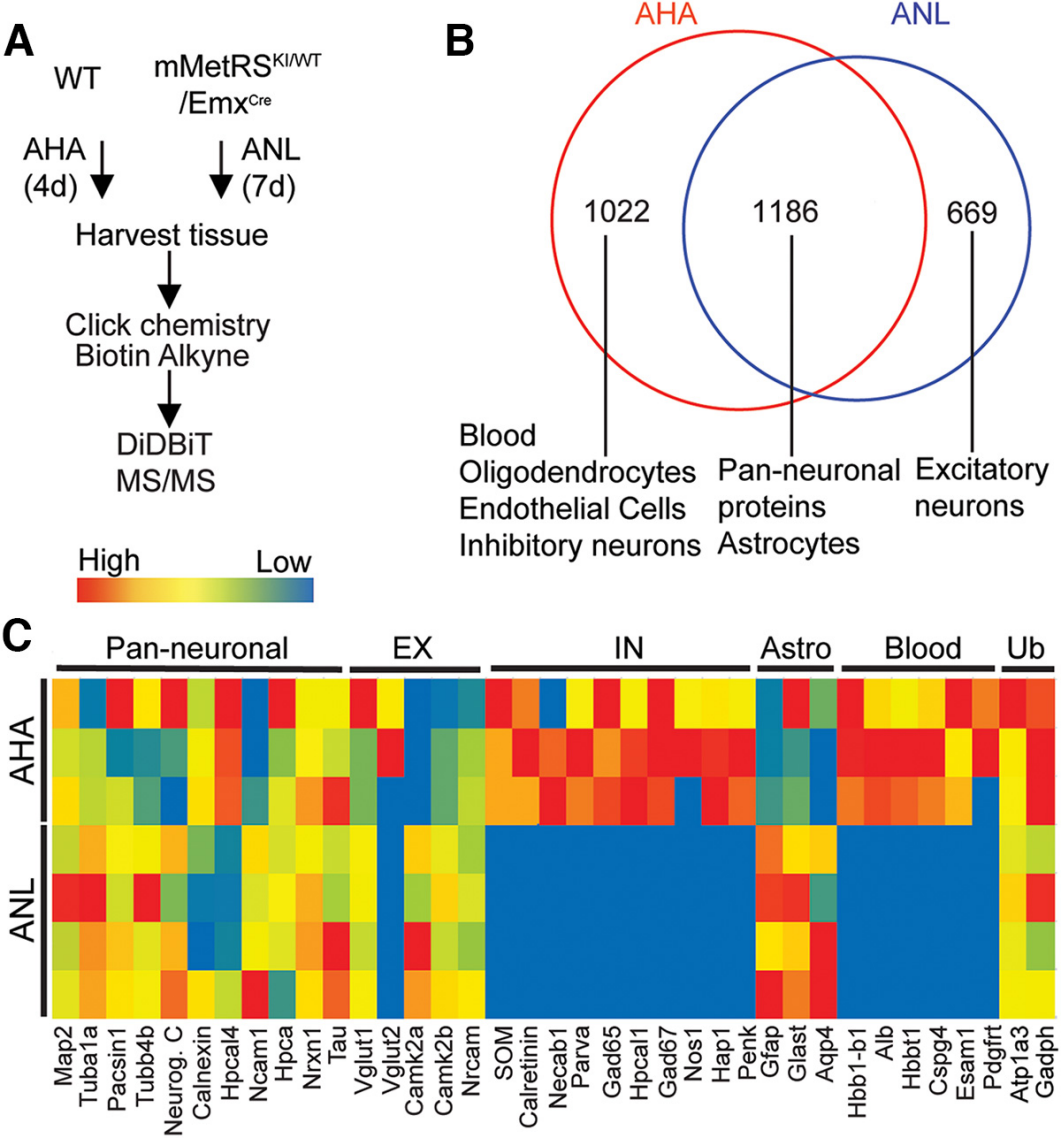
Genetic control of mMetRS expression allows identification of NSPs in specific neural cell types. ***A***, Schematic of the NSP labeling protocols for AHA and ANL. For global AHA labeling of NSPs, mice were fed AHA-laced chow *ad libitum* for 4 d. For neural cell type-specific labeling, mMetRS^KI/WT^/EMX^cre^ mice received daily intraperitoneal injections of ANL (830 mg/kg) for 1 week. For mMetRS^KI/WT^/EMX^cre^ samples, cortex was dissected from isolated brains and processed for click chemistry, DiDBiT and mass spectrometry protein identification (MS/MS). For AHA samples, the entire brain was processed for click chemistry, DiDBiT, and MS/MS. ***B***, Venn diagram showing overlap between the AHA-labeled and ANL-labeled proteomes. AHA-labeled samples include proteins from blood, vasculature (endothelial cells), oligodendrocytes, and GABAergic neurons that are absent from ANL-labeled samples. Proteins detected in both AHA-labeled and ANL-labeled samples include astrocyte and pan-neuronal proteins. Proteins uniquely labeled with ANL are annotated to excitatory neurons and astrocytes, consistent with the distributions of mMetRS expression in mMetRS/EMX^cre^ mice (Extended Data Fig. 5-1). ***C***, Heat map of relative detection of proteins from excitatory neurons, inhibitory neurons, astrocytes, blood, and ubiquitous proteins (Ub) across 4 independent MS/MS runs of samples from mMetRS^*KI/WT*^/EMX^cre^ mice (2 males and 2 females) and 3 independent MS/MS runs of AHA samples (3 males), based on spectral counts from markers of different cellular populations in ANL- and AHA-labeled proteomes (Extended Data Fig. 5-2).

To compare cell type specificity of ANL incorporation with brain-wide labeling with AHA, we administered ANL intraperitoneally daily for 7 d to mMetRS^KI/WT^/Emx^cre^ mice and collected brains the day after the last ANL treatment (Fig. 5*A*). Cortical samples were processed to label ANL-proteins with biotin-alkyne using click chemistry, and then proteins were digested with trypsin and biotin-labeled peptides were purified on NeutrAvidin beads, following the DiDBiT protocol (Schiapparelli et al., 2014). We compared the proteome results of four MS/MS runs of ANL-biotin-labeled proteins from cortex from mMetRS^KI/WT^/Emx^cre^ mice with three MS/MS runs of AHA-biotin-labeled proteins from cortices of wild-type mice fed AHA-supplemented chow for 4 d (McClatchy et al., 2015). We detected in total 1855 ANL-biotin-labeled proteins and 2208 AHA-biotin-labeled proteins (Fig. 5*B*, Extended Data Fig. 5-1). Of the 1855 ANL-labeled proteins, 1186 overlap with AHA-labeled proteins, and these proteins are annotated broadly to neurons and to astrocytes. A total of 669 proteins were uniquely detected in the ANL-labeled dataset and are likely specific to excitatory neurons. None of the proteins detected in the mMetRS^KI/WT^/Emx^cre^ ANL-biotin-labeled protein dataset were annotated to plasma, the vasculature, oligodendrocytes, endothelial cells or inhibitory neurons. Conversely, 1022 proteins were uniquely detected in the AHA-labeled dataset, including proteins found in blood, oligodendrocytes, endothelial cells, and inhibitory neurons (Fig. 5*B*,*C*, Extended Data Fig. 5-2). We plotted the relative detection of cell type marker proteins for excitatory neurons, inhibitory neurons, astrocytes, and blood for each MS/MS run in a heat map (Fig. 5*C*, Extended Data Fig. 5-2). Excitatory neuronal proteins and pan-neuronal proteins, such as Tubulin1a, Pacsin 1, Tubulin 4b, Neuroglycan C, NCAM1, VGlut1, and Neurexin1 were highly enriched in the ANL-labeled cortical proteomes from mMetRS^KI/WT^/Emx^cre^ mice, as well as the AHA-labeled cortical proteomes from wild-type mice. Interestingly, the vesicular glutamate transporter Vglut2, a marker of excitatory presynaptic terminals, was detected only in AHA-labeled cortical proteomes, but not in the cortical glutamatergic neuronal proteomes from mMetRS^KI/WT^/Emx^cre^ mice. One possible explanation for this observation is that Vglut2 detected in the cortex is expressed in thalamocortical axonal projections from thalamic neurons which do not express EMX1 (Gorski et al., 2002). In contrast to the strong representation of inhibitory neuronal proteins in the AHA-labeled proteome, proteins from inhibitory cells such as somatostatin, parvalbumin, GAD65, GAD67, pro-enkephalin, nitric oxide synthase, and calretinin are not detected in the ANL-labeled proteome from mMetRS^KI/WT^/Emx^cre^ mice, consistent with the lack of EMX1 expression in cortical inhibitory neurons (Gorski et al., 2002). Similarly, components from blood, plasma, and pericytes such as Hbb1-b1, albumin, Esam1, and Pdgrt were detected in AHA-fed animals, but not in the ANL-labeled dataset from mMetRS^KI/WT^/Emx^cre^ mice. Finally, as predicted by the EMX expression pattern, astrocyte markers, including GFAP, GLAST (glutamate–aspartate transporter), and Aquaporin4, were detected in the ANL-labeled samples. Astrocyte-specific proteins are less well detected in AHA-labeled samples compared with ANL-labeled samples (Fig. 5*C*). The spect counts for ANL and AHA samples (Extended Data Fig. 5-2) show that astrocytic markers such as GLAST (Slc1a3) and GLT (glutamate transporter; Slc1a2) are detected in AHA samples runs, although AHA samples have fewer spectrometry counts than ANL samples. This is likely because they are less prevalent in the heterogeneous protein sample from the whole brain. These results demonstrate the capacity to identify the proteome of genetically defined cell types within complex multicellular organs for specific proteomic analysis, effectively excluding contaminant proteins from the proteomic datasets.

10.1523/JNEUROSCI.0707-22.2022.f5-1 Figure 5-1 Data for Figure 5*B*. AHA versus ANL Venn diagram. Download Figure 5-1, XLSX file.

10.1523/JNEUROSCI.0707-22.2022.f5-2 Figure 5-2 Data for Figure 5*C*. AHA versus ANL heat map. Download Figure 5-2, XLSX file.

### Proteomic identification of newly synthesized proteins in cortical glutamatergic neurons after acute seizure

**Figure 6.**
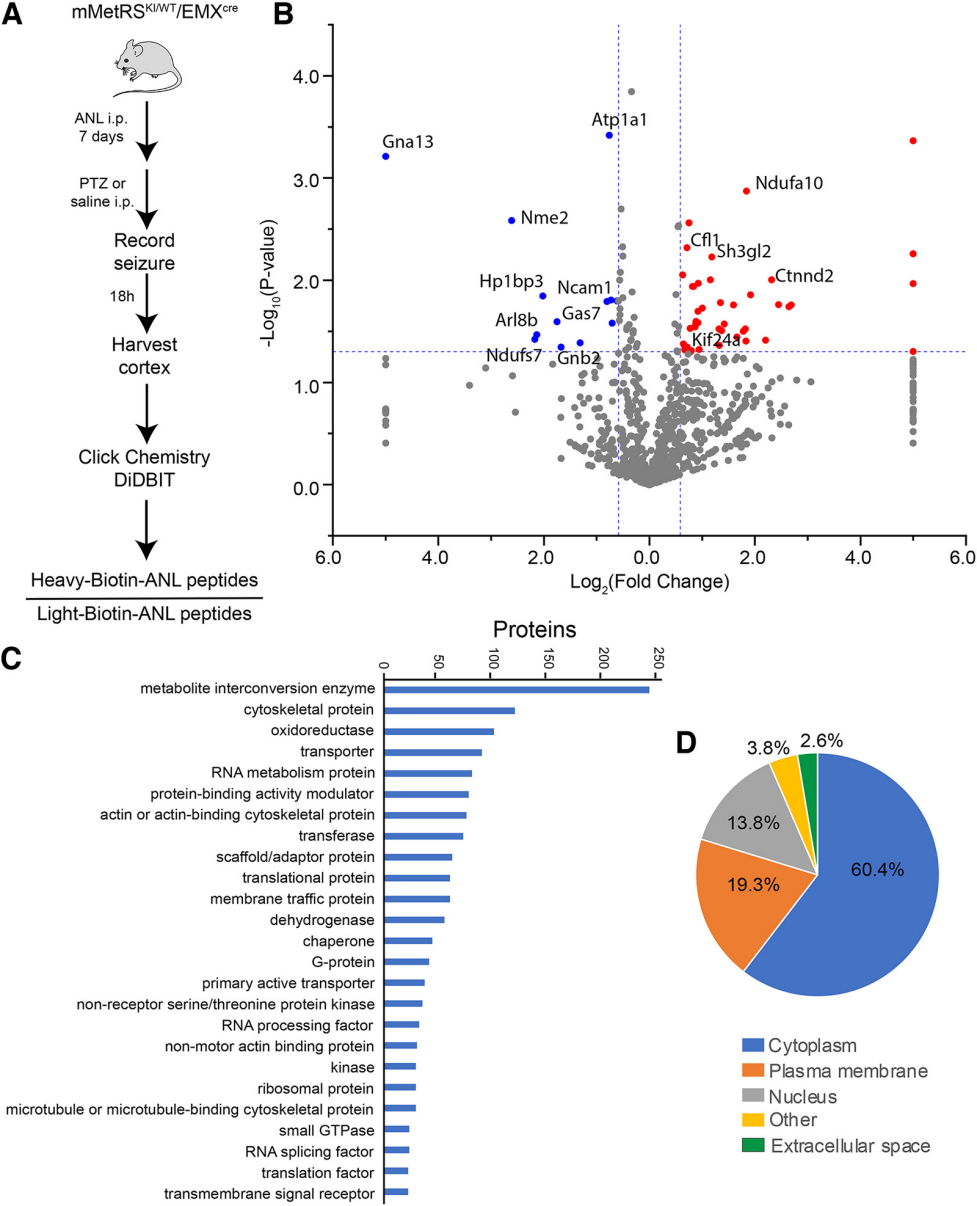
Activity-induced nascent proteomic dynamics in excitatory cortical neurons following 7 d of ANL treatment. ***A***, Schematic of experimental protocol. Adult mMetRS^KI/WT^/EMX^cre^ mice were treated with a daily dose of ANL (830 mg/kg, i.p.) for 1 week followed by a single dose of PTZ (30–39 µg/kg, i.p.) to induce seizure. The brain was harvested 18–20 h later. Cortical tissue was processed to tag ANL-containing proteins with heavy or light isotopically labeled biotin-alkyne using click chemistry before LC-MS/MS analysis of biotinylated peptides. ***B***, Volcano plot showing quantitative proteomic analysis of PTZ versus control samples. Statistically significantly (*p* < 0.05, |FC|>1.5) increases or decreases are labeled in red or blue, respectively. *N* = 4 pairs of control and PTZ-treated mice, with 3 pairs of males and 1 pair of females (Extended Data Fig. 6-1, see for list of all quantified peptides). ***C***, ***D***, Protein classes (***C***) and cellular localization (***D***) of the NSPs labeled with ANL [Extended Data Figs. 6-2 and 6-3, see for data on Top Protein Categories (Panther) and Protein Subcellular Localization (IPA)].

To identify newly synthesized proteins in cortical glutamatergic neurons induced by increased cortical activity, we induced seizure by treating mice with a single intraperitoneal injection of the GABA_A_ receptor antagonist PTZ. We administered ANL (830 mg/kg) to mMetRS^KI/WT^/Emx^cre^ mice by seven daily intraperitoneal injections over 1 week. We then administered PTZ (30–39 µg/kg, i.p.) 30 min after the final ANL injection, monitored animals for seizure, and collected cortical tissue 18 h later from animals that underwent seizure (Fig. 6*A*). Cortical samples were processed as described above to biotinylate ANL-tagged proteins using click chemistry and to incorporate heavy and light alkyne tags, which are used for our quantitative proteomics (McClatchy et al., 2015). We used heavy biotin-alkyne for the PTZ group and light biotin-alkyne for the control group, or vice versa. The samples from the two conditions were then combined, digested, and purified. Heavy and light biotin peptides were directly identified by LC-MS/MS using DiDBiT. This method decreases run-to-run variance across samples and improves statistical analysis and quantification. We identified 1315 NSPs, of which 103 were significantly different from NSPs from saline-injected controls (*p* < 0.05; Fig. 6*B*, Extended Data Fig. 6-1). Fifty-eight NSPs were significantly increased and 45 NSPs were significantly decreased in response to elevated activity. The NSPs were categorized according to protein classes using Panther (Fig. 6*C*, Extended Data Fig. 6-2), which indicated that the categories with the most NSPs were metabolic proteins and cytoskeletal proteins. Analysis of subcellular distribution of the NSPs using Ingenuity Pathway Analysis indicated that 60.4% are cytoplasmic, while 19.3% are localized to the plasma membrane and 17.8% to the nucleus. Finally, 2.6% are annotated to the extracellular space (Fig. 6*D*, Extended Data Fig. 6-3).

10.1523/JNEUROSCI.0707-22.2022.f6-1 Figure 6-1 Data for Figure 6*B*. Seven day ANL treatment PTZ versus control volcano plot from all quantified peptides. Download Figure 6-1, XLSX file.

10.1523/JNEUROSCI.0707-22.2022.f6-2 Figure 6-2 Data for Figure 6*C*. Seven day ANL treatment PTZ versus control Top Protein categories using Panther. Download Figure 6-2, XLSX file.

10.1523/JNEUROSCI.0707-22.2022.f6-3 Figure 6-3 Data for Figure 6*D*. Seven day ANL treatment PTZ versus control Protein Subcellular Localization using IPA. Download Figure 6-3, XLSX file.

**Figure 7.**
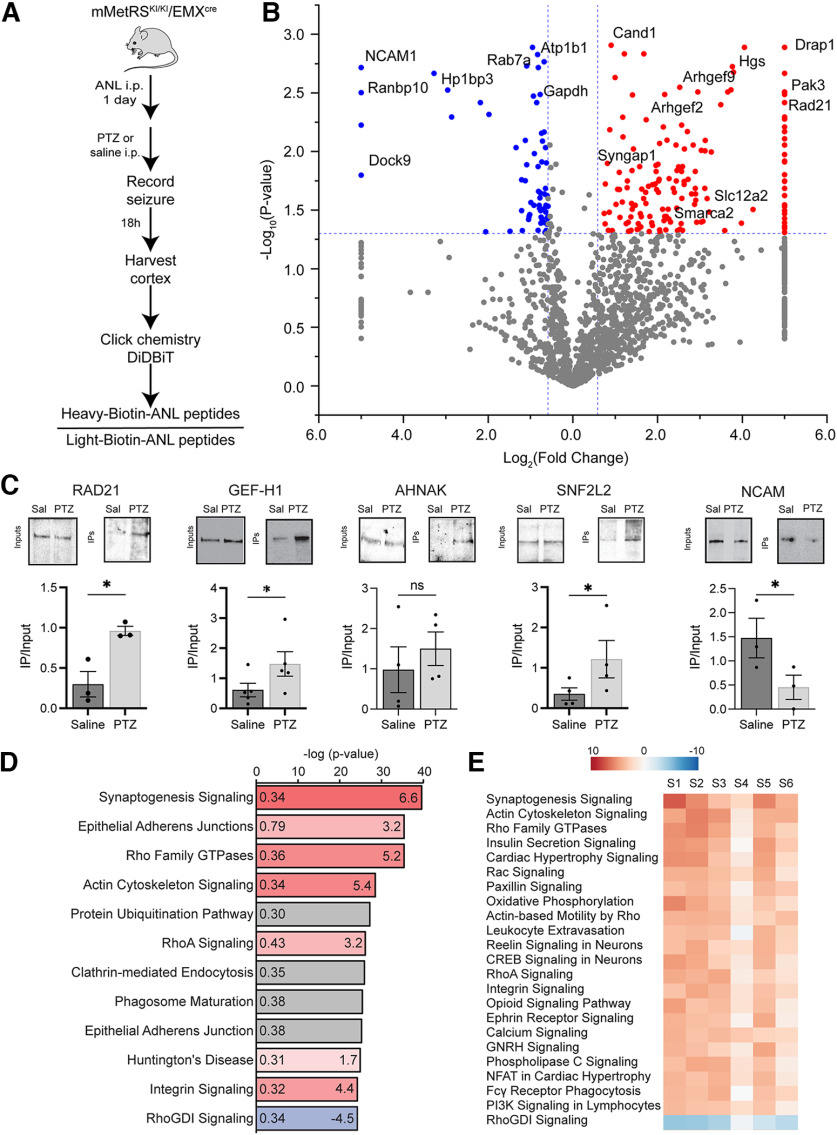
Temporal control of ANL treatment improves MS/MS detection of newly synthetized proteins in excitatory cortical neurons after acute seizure induced by PTZ. ***A***, Experimental design to identify changes in newly synthetized proteins after acute seizure. mMetRS^KI/KI^/EMX^cre^ mice received a single intraperitoneal dose of 47 μg/kg PTZ together with ANL or saline; 18–20 h later, brains were collected and cortex was isolated and processed for click chemistry with heavy and light biotin alkynes and DiDBiT to isolate biotinylated peptides for mass spectrometry protein identification. ***B***, Volcano plot showing quantitative proteomic analysis of changes in newly synthesized proteins induced by PTZ compared with saline. *N* = 4 independent experiments (3 pairs of males and 1 pair of females). Significantly increased and decreased (*p* < 0.05, |FC| > 1.5) newly synthetized proteins are labeled in red and blue, respectively (Extended Data Fig. 7-1, see for list of all quantified peptides; Extended Data Fig. 7-2, see for lists of proteins from the 7 and 1 d ANL treatment experiments). ***C***, Western blots of inputs and IPs comparing selected NSPs in saline-injected and PTZ-injected samples. Quantification of labeling: individual datapoints and mean ± SEM; *N* = 4 independent samples. *p < 0.05 Student's *t* test with Welsh's correction. n.s., not significant; sal, saline. ***D***, IPA (QIAGEN) canonical pathway analysis identified the signaling pathways that change the most in response to PTZ treatment compared with saline treatment. The color of bars represents the *z* score (values provided to right within bars) that predict increases (red) or decreases (blue) in the function of each pathway. The gray bar represents pathways where no prediction can be made (Extended Data Fig. 7-3). ***E***, Comparative analysis showing *z* scores of the most significantly changed canonical pathways across 6 independent samples (S1-S6; 3 pairs of males and 3 pairs of females; Extended Data Fig. 7-4).

The observation that biotin labeling was significantly greater in mMetRS^KI/KI^/Emx^cre^ mice than mMetRS^KI/WT^/Emx^cre^ mice (Fig. 4) suggests that we could increase the yield of NSP labeling using mMetRS^KI/KI^/Emx^cre^ mice. Furthermore, the pharmacokinetic profile of ANL in the brain (Fig. 4) suggests that we may also gain temporal resolution in the NSP labeling by collecting tissue after a single ANL injection. To identify acute changes in newly synthetized proteins in cortical glutamatergic neurons in response to increased brain activity, we induced seizure in adult homozygous mMetRS^KI/KI^/Emx^cre^ mice with a single intraperitoneal dose of the GABA_A_ receptor antagonist PTZ (47 µg/kg), delivered 30 min before a single intraperitoneal injection of ANL (830 mg/kg). Mice were monitored to ensure seizure activity, resulting in widespread c-Fos expression (Sando et al., 2012), and brains were removed 18 h later. Cortical tissue from four pairs of PTZ-treated mice and control saline-injected mice was processed for click chemistry with heavy and light biotin alkynes, respectively, followed by DiDBiT and quantitative MS/MS (Fig. 7*A*). Data from the four independent experiments were analyzed. Our results show that increased brain activity induced by PTZ acutely changed the nascent proteome, resulting in both increases and decreases in newly synthetized proteins (Fig. 7*B*, Extended Data Figs. 7-1, 7-2). We identified 3168 ANL-biotin-labeled newly synthetized proteins in the PTZ and control samples. Of these, 294 NSPs were significantly changed in PTZ-treated samples compared with saline-treated controls (*p* < 0.05; Fig. 7*B*, Extended Data Figs. 7-1, 7-2), with 196 NSPs significantly increased in response to PTZ, and 98 NSPs significantly decreased in response to PTZ compared with saline. We validated representative NSPs by Western blot (Fig. 7*C*), including NCAM, Rad21, AHNAK, GEPH1, and SMARCA2, which are also subjects of the bioinformatic analysis, presented below. These results indicate that two methodological modifications, shortening the ANL treatment period to correspond to the time of PTZ-induced increased brain activity and using mMetRS homozygote mice, significantly increased the detection of NSPs overall and, in particular, resulted in a threefold increased detection of NSPs that were significantly different between PTZ and saline conditions.

10.1523/JNEUROSCI.0707-22.2022.f7-1 Figure 7-1 Data for Figure 7*B*. One day ANL treatment PTZ versus control volcano plot from all quantified peptides. Download Figure 7-1, XLSX file.

10.1523/JNEUROSCI.0707-22.2022.f7-2 Figure 7-2 Protein lists for the 7 or 1 d ANL treatment, control and PTZ. Download Figure 7-2, XLSX file.

10.1523/JNEUROSCI.0707-22.2022.f7-3 Figure 7-3 Data for Figure 7*D*. IPA Canonical Pathway Analysis. Table lists the most significant canonical pathways. The significance values [*p*-value of overlap, represented as -log (*p*-value)] for the canonical pathways are calculated by the right-tailed Fisher's exact test. Ratio is the overlap ratio of proteins in the NSP dataset/total proteins in the pathway. The *z*-score indicates a predicted activation or inhibition of a pathway. Molecules provides a list of proteins from our dataset that are associated with a canonical pathway. Download Figure 7-3, XLSX file.

10.1523/JNEUROSCI.0707-22.2022.f7-4 Figure 7-4 Data for Figure 7*E*. IPA comparative analysis for canonical pathways. Table lists ingenuity canonical pathways and *z* scores for each experimental replicate. Download Figure 7-4, XLSX file.

**Figure 8.**
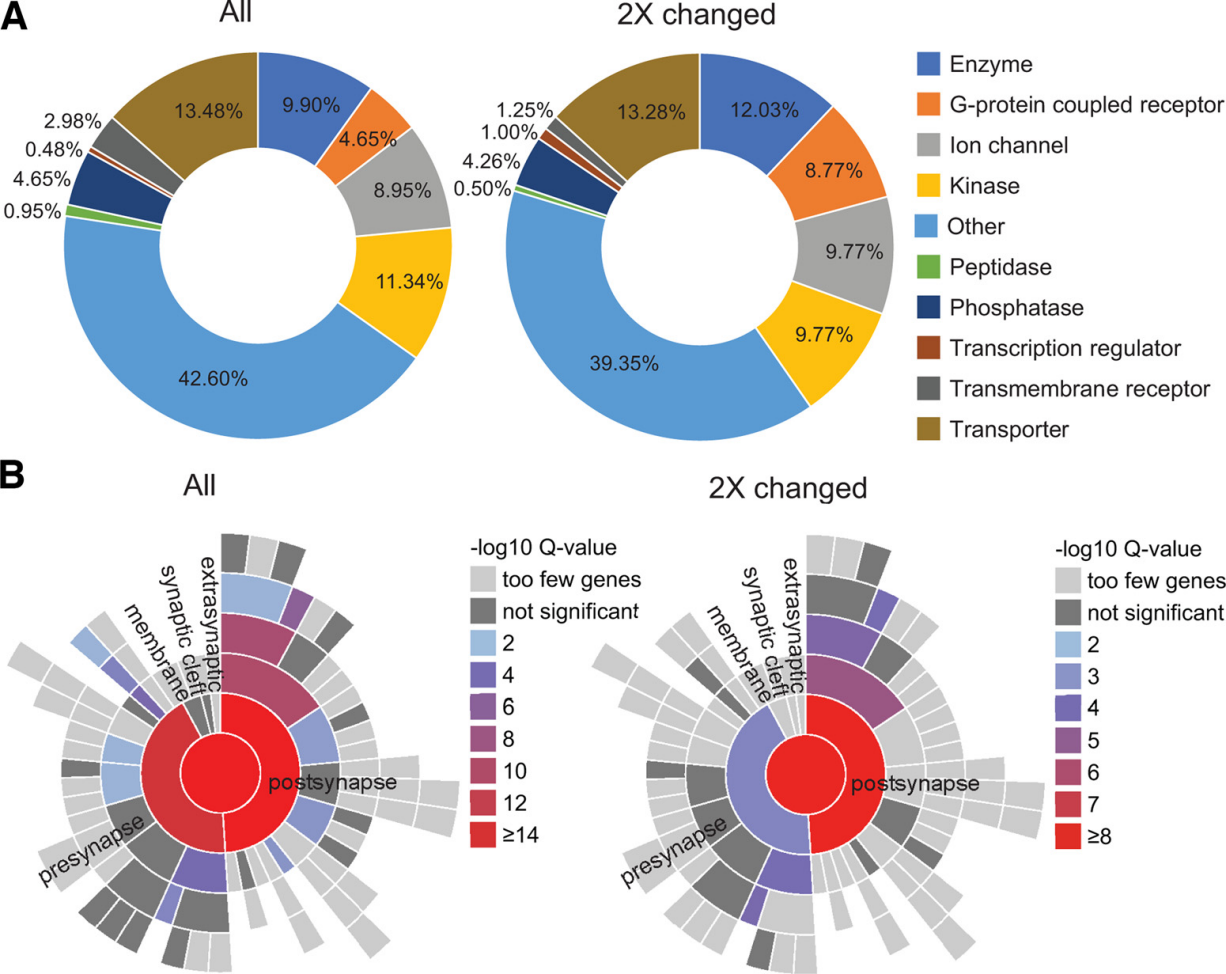
Analysis of activity-induced NSPs annotated to different subcellular compartments. ***A***, Doughnut charts showing the relative representation of functional categories of plasma membrane proteins (as annotated by QIAGEN IPA software) in the entire dataset of proteins (All, left) compared with the subset of proteins that were changed at least twofold in response to the PTZ treatment (2× changed, right). Among the more than twofold changed proteins following PTZ treatment, plasma membrane G-protein-coupled receptors and plasma membrane-associated transcriptional regulators showed the highest proportional increase. Peptidases and transmembrane receptors were decreased, and phosphatases and transporters were largely unchanged by PTZ treatment (Extended Data Figs. 8-1, 8-2). ***B***, Activity-induced synaptic NSPs are enriched in postsynaptic proteins. SynGO, a tool focused on synaptic gene ontologies, showed enrichment of diverse proteins with known synaptic functions in our dataset. The distribution of presynaptic versus postsynaptic newly synthesized proteins was heavily skewed toward postsynaptic proteins in the more than twofold changed dataset (right) compared with the entire dataset (left), indicating that PTZ treatment altered the expression of more postsynaptic proteins than presynaptic proteins (Extended Data Figs. 8-3, 8-4, 8-5, 8-6).

**Figure 9.**
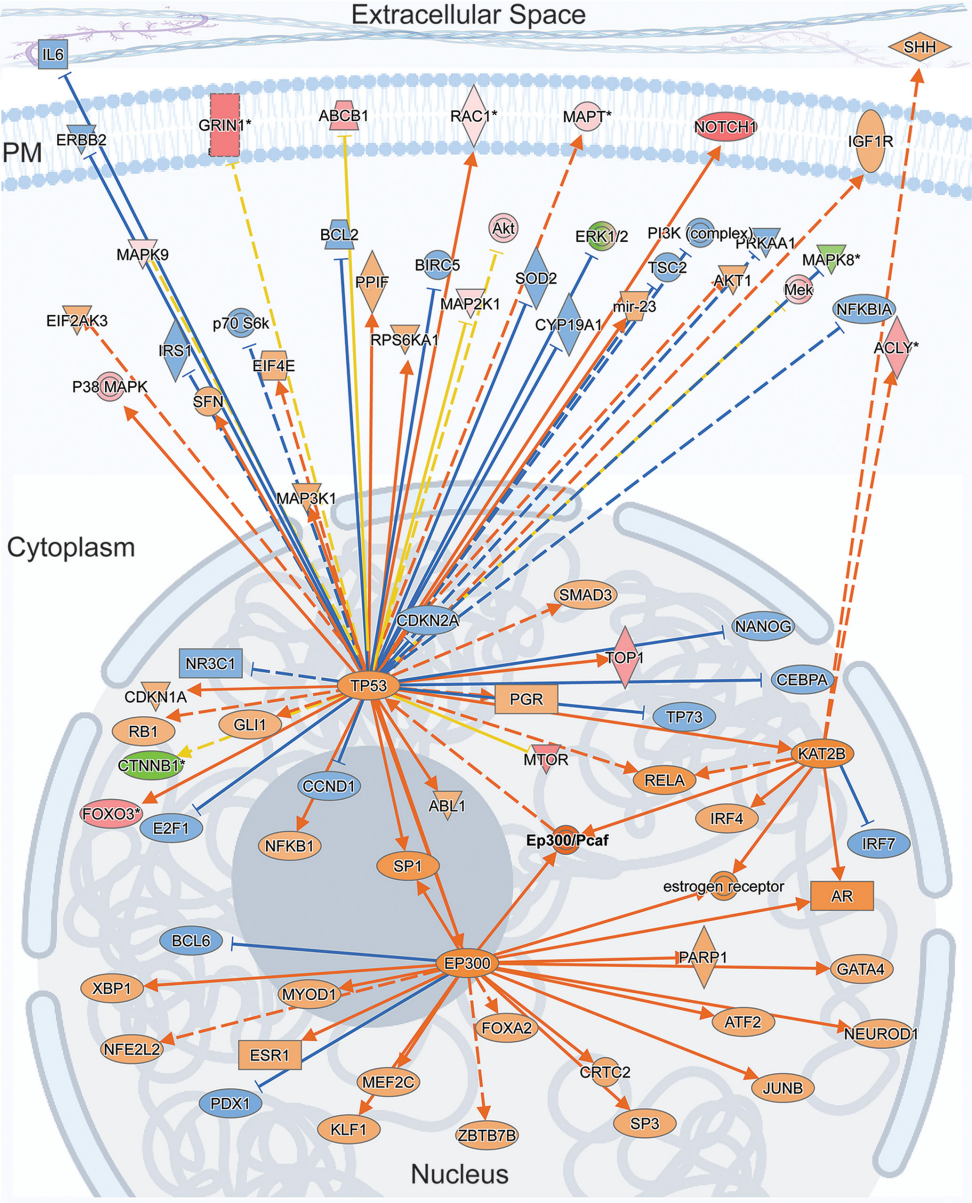
EP300/PCAF: a master regulator of activity-regulated NSPs and a possible link to long-term cellular changes via chromatin remodeling. The Ingenuity Causal Network Analysis identified the EP300/PCAF complex as the top master regulator predicted to control intermediate regulators that are responsible for protein expression changes observed in our NSP dataset. The figure shown here is a schematic of the molecular interaction network superimposed on cellular compartments, the nucleus, cytoplasm, plasma membrane, and extracellular space. TP53 (p53), EP300, and KAT2b are prominent nodes that interact with EP300/PCAF and that receive and distribute signals from a dispersed network of proteins located in the different subcellular compartments. For molecular relationships, solid lines represent direct interactions and broken lines represent indirect interactions. The arrows represent activation of targets and blocked lines represent inhibition of the targets. The orange and blue lines indicate that the relationship leads to activation or inhibition, respectively. The yellow lines indicate that the findings are inconsistent with the state of downstream molecule. The shapes and colors of the symbols indicate protein class and expression levels, respectively, as detailed in Extended Data Figure 9-4 (Extended Data Figs. 9-1, 9-2, 9-3, see for details).

**Figure 10.**
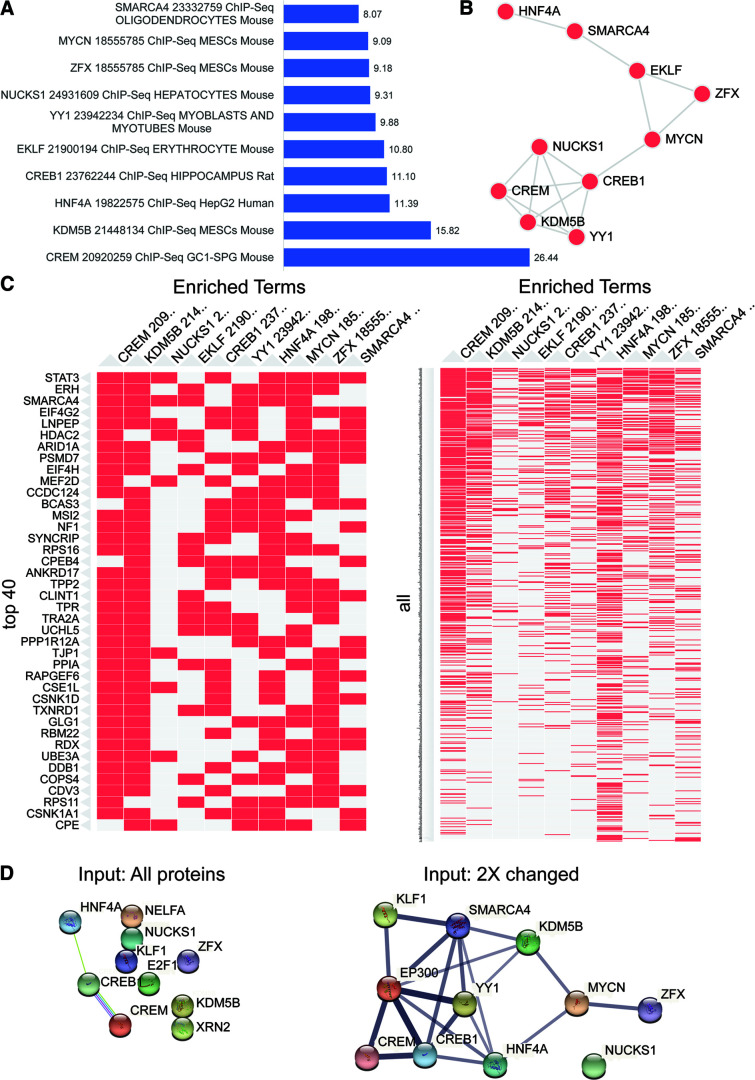
Activity-induced NSPs are enriched in ChIP-seq targets of CREB1. Enrichr transcription analysis of dataset of NSP proteins that were changed more than twofold in response to PTZ treatment. The analysis was performed using the ChEA 2016 gene set library containing functional terms representing transcription factors profiled by ChIP-seq in mammalian cells. ***A***, The top transcription factors, including information about the publication PMID number, cell type, and organism, are plotted by -log (*p*-value) shown next to the bar. ***B***, Network showing gene content similarity between the gene set libraries represented by the top transcription factors. ***C***, Clustergram showing the top 10 transcription factors (columns) with the top 40 input proteins (rows, left) or all input proteins (rows, right). The colored (red) cells in the matrix show whether the transcript of the input protein is associated with the transcription factor. ***D***, Protein–protein associations mapped using the STRING database of the top 10 transcription factors from the Enrichr analysis using all proteins (left) or the twofold changed proteins in response to PTZ (right) as the input (Extended Data Figs. 10-1, 10-2, 10-3).

In the next sections, we use several bioinformatic tools to query our NSP datasets. These analyses serve two purposes: they provide quantitative examination of our datasets, strengthening data-driven conclusions and generating hypotheses regarding downstream consequences of NSP expression (Fig. 7, Extended Data Figures 7-1, 7-2, 7-3, 7-4, Fig. 8, Extended Data Figs. 8-1, 8-2, 8-3, 8-4, 8-5, 8-6), and they predict upstream regulators of the NSPs (Fig. 9, Extended Data Figs. 9-1, 9-2, 9-3, 9-4, Fig. 10, Extended Data Figs. 10-1, 10-2, 10-3), thereby fueling hypothesis generation regarding activity-driven molecular and cellular events.

10.1523/JNEUROSCI.0707-22.2022.f8-1 Figure 8-1 Data for Figure 8*A*. IPA annotation of dataset by subcellular location and protein type. Table lists, protein symbol, Entrez gene name, subcellular location, and protein type for all NSPs. Our raw data include multiple unique peptides that could be part of the same protein. For this analysis, only unique proteins were included. Download Figure 8-1, XLSX file.

10.1523/JNEUROSCI.0707-22.2022.f8-2 Figure 8-2 Data for Figure 8*A*. IPA annotation of dataset by subcellular location and protein type. The table lists Protein Symbol, Entrez Gene Name, Subcellular location, and Protein type NSPs changed >2× compared to control. Our raw data include multiple unique peptides that could be part of the same protein. For this analysis, only unique proteins were included. Download Figure 8-2, XLSX file.

10.1523/JNEUROSCI.0707-22.2022.f8-3 Figure 8-3 Data for Figure 8*B*. SynGO analysis of all NSPs. Tables list Gene input list, gene hgnc id, gene symbol, gene name, gene synonyms, GO term ID, GO term name, GO domain, and SynGO annotation ID. Download Figure 8-3, XLSX file.

10.1523/JNEUROSCI.0707-22.2022.f8-4 Figure 8-4 Data for Figure 8*B*. SynGO analysis of NSPs changed >2× compared with control. Tables list Gene input list, gene hgnc id, gene symbol, gene name, gene synonyms, GO term ID, GO term name, GO domain, and SynGO annotation ID. Download Figure 8-4, XLSX file.

10.1523/JNEUROSCI.0707-22.2022.f8-5 Figure 8-5 Data for Figure 8*B*. SynGO ontologies for all NSPs. Tables list GO term ID, user interface reference, GO domain, GO term name, GO term name hierarchical structure, GO parent term ID, GSEA *p*-value, GSEA “gene cluster” FDR-corrected *p*-value, genes-hgnc-idm genes, hcng-symbol, and genes-your genelist input. Download Figure 8-5, XLSX file.

10.1523/JNEUROSCI.0707-22.2022.f8-6 Figure 8-6 Data for Figure 8*B*. SynGO Ontologies for NSPs changed >2× compared with control. Tables list GO term ID, user interface reference, GO domain, GO term name, GO term name hierarchical structure, GO parent term ID, GSEA *p*-value, GSEA gene cluster FDR-corrected *p*-value, genes-hgnc-idm genes, hcng-symbol, and genes-your genelist input. Download Figure 8-6, XLSX file.

10.1523/JNEUROSCI.0707-22.2022.f9-1 Figure 9-1 IPA Upstream Regulator Analysis of NSPs. IPA Upstream Regulator Analysis identifies molecules, including proteins and chemicals, that are predicted to regulate NSPs. The table lists Expr Log Ratio, Molecule Type, Predicted Activation State, Bias-Corrected *z*-score, Activation *z*-score, Flags, Bias term, *p*-value of overlap, Benjamini–Hochberg-corrected *p*-value, Target Molecules in Dataset, Mechanistic network, and Notes. Shown highlighted are 26S Proteosome (Extended Data Fig. 9-2, see details) HTT, MAPT, APP, PSEN1, and TP53. Download Figure 9-1, XLSX file.

10.1523/JNEUROSCI.0707-22.2022.f9-2 Figure 9-2 IPA Upstream Regulator Analysis indicates that the 26S Proteosome complex is inhibited by 31 target NSPs in our dataset. Table lists Protein ID, Genes in the NSP dataset, Prediction (based on measurement direction), Expr Log Ratio, and Findings. Download Figure 9-2, XLSX file.

10.1523/JNEUROSCI.0707-22.2022.f9-3 Figure 9-3 Data for Figure 9 IPA Causal Networks Analysis Identifies Master Regulators Table lists Master Regulators, including proteins, miRNAs and chemicals, Expr Log Ratio, Molecule Type, Participating Regulators, Depth, Predicted Activation or Inhibition, Notes, Activation z-score, p-value of overlap, Network bias-corrected p-value, Target Molecules in Dataset, Causal Network, Target-connected regulators. Download Figure 9-3, XLSX file.

10.1523/JNEUROSCI.0707-22.2022.f9-4 Figure 9-4 IPA Analysis of NSP Regulator Effects. IPA functional network analysis links regulation of a phenotype, function, or disease to upstream regulators. Table lists: Network ID, Consistency Score, Node Total, Regulator Total, Regulators, Target Total, Target Molecules in Dataset, Diseases and Functions Total, and Diseases and Functions. Shown highlighted are Regulators ANRT2, NRG1, SIM1, and SMARCAB1, which target 20 NSPs and are predicted to regulate functions Branching of cells and Cell Movement. Download Figure 9-4, XLSX file.

10.1523/JNEUROSCI.0707-22.2022.f10-1 Figure 10-1 Data for Figure 10. Activity-induced NSPs are enriched in ChIP-seq targets of CREB1. ChEA 2016 All Proteins. Table lists: Term (the top transcription factors, publication PMID number, cell type, and organism), -log (*p*-value), Overlap, *p*-value, Adjusted *p*-value, Odds Ratio, Combined Score, and Genes. Download Figure 10-1, XLSX file.

10.1523/JNEUROSCI.0707-22.2022.f10-2 Figure 10-2 Data for Figure 10. Activity-induced NSPs are enriched in ChIP-seq targets of CREB1. ChEA 2016 2× Changed NSPs. Table lists: Term (The top transcription factors, publication PMID number, cell type, and organism), -log (*p*-value), Overlap, *p*-value, Adjusted *p*-value, Odds Ratio, Combined Score, and Genes. Download Figure 10-2, XLSX file.

10.1523/JNEUROSCI.0707-22.2022.f10-3 Figure 10-3 Data for Figure 10*D*. Protein–protein associations mapped using the STRING database of the top 10 transcription factors from the Enrichr analysis using the twofold changed proteins in response to PTZ as the input. Table lists: Nodes 1,2, Nodes 1, 2 STRING ID, coexpression, experimentally determined interaction, database annotated, automated text mining, and combined score. Download Figure 10-3, XLSX file.

### Neuronal activity driven protein synthesis dynamics reinforce signaling cascades involved in synaptogenesis

Activity-induced protein synthesis is widely recognized as a mechanism to reinforce neuronal plasticity at the level of cells as well as synapses. As such, activity-dependent proteome dynamics are expected to reveal changes in NSPs involved in synaptic plasticity, as well as maintenance and stabilization of plastic synapses. While increased neuronal activity is known to trigger robust changes in protein synthesis, details about the regulation, scope and purpose of activity-induced NSP dynamics are still unclear. Bioinformatic pathway analysis is a valuable tool to investigate the signaling and regulatory cascades that are triggered and reinforced by these increases and decreases in new protein synthesis that are induced by increased neuronal activity.

We used IPA software (QIAGEN) to annotate the entire dataset of NSPs and the subset of 813 NSPs that were changed at least twofold in response to PTZ and perform canonical signaling pathway analysis. IPA predicts signaling pathways that change based on differential synthesis of NSPs in response to activity. The Synaptogenesis Signaling Pathway was the top canonical pathway identified by IPA, with an activation *z* score of 6.6 and an overlap ratio of 0.34, meaning that 34% of proteins in our dataset were mapped to the canonical pathway (Fig. 7*D*, Extended Data Fig. 7-3). This shows that Synaptogenesis Signaling is the most robust function mediated by proteins whose synthesis increased or decreased in response to neuronal activity. Most of the other top canonical signaling pathways identified by IPA are also related to neuronal and synaptic function, including CREB Signaling in Neurons, Cytoskeletal Rearrangements (Rho signaling, Actin, Rac signaling), Remodeling of Cell Adhesion Molecules (Integrins, Adherens Junction Remodeling and Signaling), Clathrin-Mediated Endocytosis, Huntington Disease Signaling, Ephrin Signaling, Reelin Signaling in Neurons, and Calcium Signaling (Fig. 7*D*). We also conducted a comparative analysis of canonical signaling pathways across the experimental replicates. Using the *z* score, a relatively strict criterion to compare across canonical pathways, we find that all the experiments showed the same top pathways with same direction of *z*-score change (Fig. 7*E*, Extended Data Fig. 7-4). Together, this analysis indicates that activity-induced changes in NSPs in cortical glutamatergic neurons affect components of diverse signaling pathways related to gene expression, cellular interactions, and neuronal and synaptic remodeling.

### Analysis of NSPs annotated to different subcellular compartments

Analysis of activity-induced changes in NSPs annotated by different subcellular compartments and by protein class may resolve functionally relevant changes in NSPs and foster testable hypotheses regarding activity-induced dynamics of specific NSPs. We used IPA software to compare the proportions of NSPs annotated to different subcellular localizations in the entire dataset of NSPs with the dataset of NSPs that were changed two or more times by increased activity. This analysis revealed comparable overall distributions in the two datasets (Extended Data Figs. 8-1, 8-2), indicating that increased activity alters NSP expression widely across subcellular locations. By contrast, when plotted by protein type, GPCRs doubled their representation from 0.81% to 1.59% in the dataset of proteins that was significantly changed by increased activity (Extended Data Figs. 8-1, 8-2). Combining subcellular localization with protein type provided deeper insight. Among the NSPs with a more than twofold change following PTZ treatment, plasma membrane G-protein-coupled receptors and plasma membrane-associated transcriptional regulators showed the highest proportional increases. Peptidases and transmembrane receptors were decreased, and phosphatases and transporters were largely unchanged by PTZ treatment (Fig. 8*A*, left, All NSPs, right, 2× changed, Extended Data Figs. 8-1, 8-2).

Synaptic plasticity and modulation are primary functions of activity-dependent downstream processes. We were therefore interested in whether activity-regulated NSPs include synaptic proteins. We used SynGO, a public knowledgebase of synaptic genes/proteins compiled from published experimental data (Koopmans et al., 2019), to identify proteins in our dataset that are annotated to synapses and map their predicted localization in presynaptic and postsynaptic compartments (Fig. 8*B*, Extended Data Figs. 8-3, 8-4, 8-5, 8-6). Four hundred fifty NSPs in our entire dataset mapped to SynGO annotation of synaptic proteins with the filtering stringency set to high (Fig. 8*B*, left, all NSPs). Of these, 241 NSPs were annotated by “Cellular Component,” which categorizes proteins according to the subsynaptic location where they are active; 235 NSPs were annotated to “Biological Process,” which categorizes the proteins according to the synaptic processes that they carry out. The annotations can be overlapping. Among the NSPs that changed two or more times in response to increased activity, 156 NSPs showed SynGO annotations, with 91 NSPs annotated by Cellular Component and 86 NSPs annotated by Biological Processes (Fig. 8*B*, right, two times changed). We generated a map of gene set enrichment analysis (GSEA) *p*-values for synaptic cellular components and found that the dataset including all NSPs showed comparable *p*-values for presynapse and postsynapse hierarchical annotations (Fig. 8*B*, left). By contrast, in the NSPs that were changed more than two times in response to increased activity, postsynaptic proteins were more significantly enriched than presynaptic proteins (Fig. 8*B*, right). While validating these results, for instance using immunoprecipitation of biotin-tagged synaptic NSPs from cell type-specific synaptosome preparations from cortical tissue from mMetRS^KI/KI^ mice treated with ANL and PTZ, followed by Western blot labeling for NSP candidates, would be technically challenging, this bioinformatic analysis nonetheless suggests that increased activity leads to more robust and significant changes in the expression of NSPs that are specifically annotated to postsynaptic sites compared with presynaptic sites. As recent and future studies expand neuroproteomic datasets and increase their resolution with respect to subcellular compartments, biological and experimental conditions, and genetically identified neural cell types, predictive analysis of the subcellular distribution of proteins in neuroproteomic datasets, including synaptic distribution, will likely become more complete.

### Activity-induced NSP dynamics reveal noncanonical upstream regulators that bridge traditional synaptic proteins and transcription

Identifying the regulatory proteins that are responsible for the activity-induced changes in NSPs may help us understand how activity orchestrates signaling networks to modulate synaptic function. To identify transcriptional regulators, we used upstream transcription regulator analysis of QIAGEN IPA software to identify transcription factors, and their regulators predicted to lead to our observed changes in NSPs. We used strict criteria in the analysis, in which only experimentally observed relationships (not predicted binding) between regulators and NSPs were used to predict upstream transcriptional regulators. MAPT, APP, TP53, PSEN1, and HTT were identified as the top five upstream regulators (ranked by *p*-value; Extended Data Fig. 9-1, Upstream Regulators) that were predicted to regulate the protein expression changes we observe in our activity-dependent NSP dataset, consistent with recent studies indicating a role for MAPT and other plasma membrane-associated proteins in transcriptional regulation (Evans et al., 2019; Paudel et al., 2020; Montalbano et al., 2021). MAPT/TAU is an interesting example of a protein with multiple potential activity-regulated interactions. It is well-studied in the context of neurodegenerative diseases and has a known role in destabilizing synapses in pathogenic conditions (Zhou et al., 2017; Paudel et al., 2020). More interestingly, the expression of Tau itself is regulated by activity, and Tau has functions in regulating mRNA transcription and alternative splicing (Kobayashi et al., 2019; Montalbano et al., 2021). Similarly, APP, TP53, PSEN1, and HTT have all been shown to have critical roles in synaptic function. This analysis suggests that mechanistically these proteins may influence both transcription and synaptic function at least partially through effects on activity-induced changes in NSPs.

Recent studies identified cell adhesion molecules that can affect protein expression by regulating transcription factors (Kozlova et al., 2020). Using the analysis mentioned above to identify categories of NSPs within subcellular compartments, we found that transcriptional regulators associated with plasma membrane were relatively enriched in our dataset of activity-regulated NSPs (Fig. 8*A*). For example, the multifunctional sterol regulatory-element binding proteins (SREBPs) SREBF1/2 are upstream regulators identified by IPA upstream regulator analysis (Extended Data Fig. 9-1). SREBPs are plasma membrane-associated transcription factors that mediate rapid control of transcription of genes associated with lipid metabolism and cholesterol homeostasis, and also directly regulate signaling pathways pertaining to cell growth (Shimano and Sato, 2017). SREBF1/2 are basic helix-loop-helix-leucine zipper (bHLH-Zip) transcription factors that bind to sterol regulatory element-1 (SRE1) to regulate transcription of sterol-regulated genes. In neurons, SREBPs reportedly control dendrite development (Vik and Rine, 2000; Ziegler et al., 2017). Together, these examples demonstrate how IPA can identify noncanonical candidate upstream mechanisms by which neuronal activity may generate dynamics in the nascent proteome of glutamatergic neurons.

### Activity-dependent regulation of 26S proteasome activity

The 26S proteasome is a multiprotein complex involved in the ATP-dependent degradation of ubiquitinated proteins. It is widely recognized that ubiquitin-proteasomal regulation of proteostasis is essential for activity-dependent neuronal plasticity and is engaged in response to seizure (Tai and Schuman, 2008; Hamilton and Zito, 2013; Liu et al., 2021; Türker et al., 2021). We see significant increases in Ube4b, Ube2h, Ddb1, Cand1, and Cullin 3, which are components of E3 protein ubiquitination ligase pathways in response to increased neuronal activity, possibly contributing to homeostatic responses with activity-dependent increased protein synthesis (Lignani et al., 2020). Applying IPA upstream regulator analysis to our dataset predicted 26S proteosome function to be significantly reduced by PTZ treatment with a bias-corrected activation *z* score of −2.653 (Extended Data Fig. 9-2). The IPA regulator analysis identified 31 proteins in our dataset of changed NSPs, including PSAP, FOXO1, NOTCH1, PSMC2, SQSTM1, GRIN1, GRIA1, VIM, WBP2, GFAP, PSMB5, and PRNP, which were linked to a reduction of 26S proteasome activity (Extended Data Fig. 9-2). Some of these proteins are components of 26S proteasome.

### EP300/PCAF complex: a master regulator of activity-regulated NSPs and possible link to long-term cellular changes via chromatin remodeling

To further identify potential upstream regulators of the PTZ-induced NSPs, we applied the QIAGEN IPA causal network analysis to our datasets. IPA causal network analysis is a method of predicting upstream regulators based on causal relationships and allowing multiple layers of regulation of gene/protein expression changes (Krämer et al., 2014). It can identify potential master regulators of gene/protein expression in datasets. This analysis identified EP300/PCAF as one of the top master regulators that can orchestrate the protein expression changes we observe via multiple layers of regulation, as schematized in Figure 9. EP300/PCAF is predicted to regulate a wide array of regulators like BCL2, ERK1/2, Estrogen Receptor, GRIN1, MAPT, MTOR, NANOG, NEUROD1, NOTCH, and SHH, which converge onto TP53, KAT2B, or EP300 (Fig. 9, Extended Data Fig. 9-3). EP300 is a transcriptional coactivator that functions as a histone acetyltransferase and regulates transcription via chromatin remodeling (Ogryzko et al., 1996) of genes involved in synaptogenesis, synaptic plasticity, memory formation, and maintenance of neuronal identity (Maurice et al., 2008; Fischer, 2014; Guzman-Karlsson et al., 2014; Tapias and Wang, 2017; Lipinski et al., 2020). In addition, EP300 is involved in RNA splicing (Rahhal and Seto, 2019), supporting its central roles in neuronal development and plasticity. SMARCA2, one of the downstream targets of EP300 in our dataset (Fig. 7*C*, Extended Data Fig. 9-3), is a member of the SWI/SNF (Switch/Sucrose Non-Fermentable) family of chromatin remodeling proteins that is involved in transcriptional activation and repression.

To connect these regulatory signaling cascades with biological functions, we applied IPA functional network analysis, which can identify how a phenotype, function, or disease is regulated in the dataset by upstream regulators. We identified four transcription factors, ARNT2, NRF1, SIM1, and SMARCB1, which are predicted to regulate target proteins in our dataset associated with IPA categories of biological functions relevant to morphologic dynamics and signaling pathways: branching of cells, cell movement of fibroblast cell lines, protein kinase cascade, shape change of neurites, shape change of neurons, and sprouting (consistency score, 8.05). The target NSPs regulated by these upstream regulatory transcription factors include nuclear proteins and synaptic proteins (Extended Data Fig. 9-4), consistent with the significant activity-induced changes in synaptic proteins (Fig. 8) and chromatin-remodeling proteins SMARCA2, Rad21, and Chd4, which are increased ∼20-fold in response to PTZ-induced activity (Fig. 7*C*, Extended Data Figs. 7-1, 7-2). Together, these analyses, using IPA causal network analysis and IPA functional network analysis, suggest that the EP300/PCAF complex together with the SMARCA SWI/SNF complex may be a link between the orchestration of activity-regulated nascent proteomic changes and long-term cellular consequences via chromatin remodeling.

### Activity-induced NSPs are enriched in ChIP-seq targets of CREB1

Enrichr transcription factor analysis offers an independent strategy to identify transcription factors that could regulate our activity-dependent NSPs. Enrichr is a GSEA tool developed to test whether a dataset is enriched with genes/proteins that are putative transcription factor targets. It uses ChEA gene set libraries containing functional terms representing transcription factors profiled by published chromatin immunoprecipitation with massively parallel DNA sequencing (ChIP)-chip, ChIP-seq, ChIP-PET, and DamID experiments. We used the ChEA 2016 gene set library containing functional terms representing transcription factors profiled by ChIP-seq in mammalian cells. MYCN, HNF4A, CREB1, and SMARCA4 are among the top 10 transcription factor hits (Fig. 10*A*,*C*, Extended Data Figs. 10-1, 10-2), and these proteins also appear prominently in the IPA of upstream regulators (Extended Data Figs. 9-1, 9-2, 9-3, 9-4). A network of gene content similarities between the gene set libraries represented by the top 10 transcription factors (Fig. 10*B*) highlights the interactions and overlap between these regulators. Furthermore, evidence-based analysis using STRING indicates that the top 10 transcriptional regulators identified by Enrichr (Fig. 10*A*) form an interactive network based on protein–protein interactions of the activity-induced NSPs (Fig. 10*D*, Extended Data Fig. 10-3). Of note, EP300, identified by IPA as one of the master regulators, is part of this interaction network of transcription factors. Even with evidence-based analysis with the highest confidence score (interaction score, >0.9), EP300 directly interacts with CREB1, YY1, SMARCA4, and CREM (Fig. 10*D*). This independent analysis predicts that EP300 regulates a network of transcription factors to bring about the protein expression changes we observe as activity-dependent NSPs.

## Discussion

We optimized a pipeline for unbiased proteomic discovery of NSPs in genetically defined neurons and applied it to identify NSPs synthesized in mouse cortical glutamatergic neurons *in vivo* within 20 h after PTZ-induced seizure. We used HEK cells to establish that labeling NSPs with AHA or ANL is comparable. We showed that driving mMetRS expression with EMX^cre^ does not grossly affect brain structure or behavioral plasticity. We optimized the temporal resolution of labeling activity-induced NSPs *in vivo* based on the pharmacokinetic profile of intraperitoneally delivered ANL in the brain. We tagged ANL-labeled NSPs with heavy or light biotin-alkyne and processed samples with DiDBiT by tandem mass spectrometry. These technical improvements increased detection of activity-induced NSPs, and increased the reproducibility and statistical analysis of datasets. Our quantitative analysis identified ∼300 significantly differentially expressed NSPs in genetically identified cortical excitatory neurons within 20 h after seizure versus controls. We validated a selection of NSPs and applied bioinformatic tools to our datasets to add depth to our analysis and to exemplify strategies for hypothesis generation. Unlike analysis of differentially expressed RNA intermediates, we directly determined cell type-specific proteomic outcomes in response to increased activity. These activity-induced NSP dynamics are the most direct representation of downstream effectors that mediate plasticity in different neuronal compartments, including chromatin remodeling, structural changes in dendrites and axons, and synaptic functions. We hypothesize that activity-induced changes in the SMARCA SWI/SNF complex result in chromatin modifications, which work in concert with the EP300/Pcaf complex to execute activity-dependent changes in wide array of signaling networks, resulting in large-scale short-term and long-term changes. Our datasets, which are available for further analysis, and our enriched bioinformatic analyses may help to generate further hypotheses concerning the functions of activity-induced NSPs. We anticipate that this strategy has sufficient depth to identify changes in NSPs in genetically defined cell types following changes in neuronal activity, such as occurs in response to sensory stimulation.

### Activity-induced NSPs

We detected approximately threefold more significantly different PTZ-induced NSPs when we paired acute intraperitoneal ANL delivery with PTZ-induced seizure compared with the 7 d ANL treatment protocol. This increased detection may be because of both using mMetRS^KI/KI^ mice and greater temporal coincidence of elevated brain ANL and seizure. Our previous experiments indicated that visual experience significantly increased and decreased NSPs in *Xenopus* optic tectum (Liu et al., 2018). Here, ∼33% of the significantly changed NSPs decreased with PTZ treatment compared with controls. Together, these results indicate that increased activity leads to increases and decreases in specific NSPs. Decreased NSPs may arise by decreasing transcript levels (Raab-Graham and Niere, 2017; Dong et al., 2020), for instance, downstream of activity-induced expression of transcriptional repressors (Ashok et al., 2020; Dong et al., 2020), by miRNA-mediated mechanisms (Henshall et al., 2016; Bencurova et al., 2021; Zolboot et al., 2021) or by activity-induced degradation of NSPs. Recent studies identified a neuronal membrane-associated proteosome that degrades activity-induced NSPs (Ramachandran and Margolis, 2017; Ramachandran et al., 2018; He et al., 2022) and is required for behavioral plasticity (He et al., 2022). Although proteomic analysis of human brain samples indicates that seizure can increase and decrease proteins (Xiao et al., 2020), our data specifically show that activity-induced decreases in NSPs contribute to this outcome. NCAM1 was among the NSPs that were significantly decreased in response to increased activity. The decreased synthesis of NCAM in response to neuronal activity supports a model in which transsynaptic complexes are disrupted during seizures and that adhesion molecules could be cleaved by extracellular proteases, contributing to epileptogenesis (Gorlewicz and Kaczmarek, 2018). Indeed, we have recently shown that activity-dependent increased levels of the extracellular metalloprotease, MMP9, which targets the extracellular matrix (ECM) and enhances synaptic plasticity (Kaliszewska et al., 2012), are required for experience-dependent structural and functional visual circuit neuronal development (Gore et al., 2021). These examples show how activity-dependent increases and decreases in NSPs contribute to a unified biological outcome regarding modifications of the ECM.

### Synaptic protein changes and homeostasis

Examining individual NSPs associated with synapses revealed increased synthesis of GEPH1, Pak2, Pak3, and SynGAP1. These proteins are involved in trafficking AMPA receptors and the regulation of dendritic remodeling, spine development, and plasticity. Pak serine/threonine kinases are involved in cytoskeletal rearrangements and could be involved in neuronal structural rearrangements observed after seizures (Cavazos et al., 1991; Boda et al., 2004; Teskey et al., 2006; Zeng et al., 2009; Hussain et al., 2015; Crino, 2019). Regulation of SynGAP1, a component of the postsynaptic density that is critical for cortical development and cognitive function (Vazquez et al., 2004; Kang et al., 2009; Creson et al., 2019; Gamache et al., 2020; Llamosas et al., 2020), is consistent with activity-induced synaptic plasticity. Our bioinformatic analysis combining subcellular localization of NSPs with protein type indicated that GPCRs were significantly decreased among NSPs. GPCRs constitute the largest family of cell surface receptors (>800 proteins) and are critical in sensing the cellular environment. GPCRs play a key role in neuronal function by responding to diverse neurotransmitters and neuromodulators and by modulating synaptic transmission (Smith et al., 2019). The greater representation of GPCRs among the activity-responsive NSPs suggests a targeted effect on GPCRs that is critical for synaptic function and plasticity.

Seizures evoke a variety of homeostatic responses (Lignani et al., 2020). Several proteins induced by PTZ may play homeostatic roles. For instance, we found increased synthesis of the Na^+^-K^+^-2Cl^–^ cotransporter NKCC1, which regulates intracellular Cl^–^ and is required for hyperpolarizing GABAergic transmission (Wright, 2009). NKCC1 has been implicated in epilepsy, and NKCC1 protein levels are increased following seizure. Consequently, NKCC1 is a candidate target for antiepileptic drugs (Delpire et al., 2016; Liu et al., 2019). Arghef9, also called Collybistin, is robustly increased by activity. Collybistin is a scaffold protein that is required for inhibitory synapse formation and plasticity (Papadopoulos and Soykan, 2011) by clustering the GABAergic postsynaptic organizer Gephryn and GABA receptors subunits (Körber et al., 2012). As a third example of an activity-induced homeostatic protein, synthesis of AHNAK, a large scaffolding protein, is significantly increased. AHNAK interacts with voltage-sensitive L-type Ca^2+^ channels in the plasma membrane and has been shown to play an essential role in calcium homeostasis (Sundararaj et al., 2021), suggesting that increased AHNAK may have homeostatic functions following PTZ-induced neuronal depolarization.

Activity-regulated proteasome activity may contribute to homeostasis. We find increased synthesis of several components of E3 ligase pathways, whereas chemical long-term depression caused a prolonged decrease in activity of the ubiquitin–proteasome system (Tai et al., 2010), consistent with studies showing that blocking action potentials with TTX decreased ubiquitin proteasome system activity (Jakawich et al., 2010). Our bioinformatic analysis using IPA upstream regulator analysis suggests that protein degradation mechanisms and new protein synthesis are linked in a regulatory network. Because neuronal proteastasis is expected to differ between cellular compartments, our studies could not capture spatially resolved effects of activity on proteasomal control. The regulation of proteastasis in the context of activity state, mechanisms of degradation, and subcellular location of action remain poorly understood (Tai et al., 2010; Herbst et al., 2021; Soykan et al., 2021; Türker et al., 2021).

### Upstream transcriptional regulation and chromatin remodeling NSPs

NSPs provide input datasets to identify upstream transcriptional regulators responsible for activity-induced changes in NSPs, such as the master regulators EP300/Pcaf and CREB. We also found that EP300 is a master regulator in events controlling neural progenitor fate transitions and neurogenesis (Huang et al., 2021), highlighting its broad regulatory status in cellular transitions. EP300 binds CREB and other predicted upstream transcriptional regulators of our NSP dataset. NSPs themselves include proteins that regulate chromatin and subsequent waves of transcription. For instance, the NSP SMARCA2 is a member of the SWI/SNF complex, which regulates chromatin conformation, transcription factor accessibility, and gene transcription (Mashtalir et al., 2018). Interestingly, mutations in SMARCA2 have been associated with neurodevelopmental disorders (Koga et al., 2009; Wolff et al., 2012). Another NSP, Rad21, is a cohesion complex component that is involved in sensory experience-induced chromatin remodeling during motor learning (Yamada et al., 2019). Chromatin conformation changes during neuronal activity have an impact on transcription factor accessibility of proteins such as Chd4 (Yang et al., 2016), a chromatin-remodeling protein required for neurodevelopmental progression (Goodman et al., 2020). Interestingly, our quantitative proteomic analysis showed an ∼20-fold increase in newly synthesized Chd4, indicating that seizure-induced increased activity increased *de novo* synthesis of chromatin remodelers such as Rad21 and SMARCA2, as well as transcription factors, which may have greater access to DNA following chromatin remodeling. The cohesion complex and Chd4 interact to increase chromatin accessibility to transcription factors and enhancer activity (Goodman et al., 2020). Together, these analyses using IPA causal network analysis and IPA functional network analysis suggest that the upstream EP300/PCAF regulatory complex together with the activity-induced SMARCA SWI/SNF complex link orchestration of activity-regulated nascent proteomic changes and long-term cellular consequences via chromatin remodeling. This bioinformatic analysis, starting from the NSP pool, provides both a predictive retrospective assessment of upstream events, as well as prospective downstream events, which can be compared across experimental datasets and examined with additional experiments and modeling.
